# Blockade of CD93 in pleural mesothelial cells fuels anti-lung tumor immune responses

**DOI:** 10.7150/thno.89144

**Published:** 2024-01-01

**Authors:** Chengyan Zhang, Xi Nan, Bei Zhang, Hao Wu, Xianchang Zeng, Zhengbo Song, Shumin Li, Jiaoli Wang, Shaofang Xie, Gensheng Zhang, Huiqing Xiu, Jianli Wang, Jufeng Guo, Pingli Wang, Zhijian Cai, Yunfang Zhen, Yingying Shen

**Affiliations:** 1 Laboratory of Cancer Biology, Key lab of Biotherapy in Zhejiang, Cancer Center of Zhejiang University, Sir Run Run Shaw Hospital, Medical School of Zhejiang University, Hangzhou, Zhejiang 310020, China.; 2 Institute of Immunology and Department of Orthopaedics of the Second Affiliated Hospital, Zhejiang University School of Medicine, 310009 Hangzhou, China.; 3 Gastroenterology, the Second Affiliated Hospital and Yuying Children's Hospital of Wenzhou Medical University, 325000 Wenzhou, China.; 4 Department of Medical Oncology, Zhejiang Cancer Hospital, 310022 Hangzhou, China.; 5 Department of Respiratory and Critical Care Medicine, the Second Affiliated Hospital of Zhejiang University School of Medicine, 310003 Hangzhou, China.; 6 Key Laboratory of Clinical Cancer Pharmacology and Toxicology Research of Zhejiang Province, Affiliated Hangzhou First People's Hospital, School of Medicine, Westlake University, 310006 Hangzhou, China.; 7 Zhejiang University Cancer Centre, 310006 Hangzhou, China.; 8 School of Life Science, Westlake University, 310024 Hangzhou, China.; 9 Department of Critical Care Medicine of the Second Affiliated Hospital, Zhejiang University School of Medicine, 310003 Hangzhou, China.; 10 Institute of Immunology and Bone Marrow Transplantation Center of the First Affiliated Hospital, Zhejiang University School of Medicine, 310058 Hangzhou, China.; 11 Institute of Hematology Zhejiang University & Zhejiang Engineering Laboratory for Stem Cell and Immunotherapy, 310006 Hangzhou, China.; 12 Department of Breast Surgery, Affiliated Hangzhou First People's Hospital, School Of Medicine, Westlake University, 310006, Hangzhou, China.; 13 Department of Orthopaedics, Children's Hospital of Soochow University, 215002 Suzhou, China.

**Keywords:** CD93, Anti-CD93, Lung tumors, Pleural mesothelial cells, Extracellular vesicles, CCL21

## Abstract

**Background:** CD93 reportedly facilitates tumor angiogenesis. However, whether CD93 regulates antitumor immunity remains undeciphered.

**Methods:** Lung tumor tissues, malignant pleural effusions (MPEs) were obtained from lung cancer patients. Blood was obtained from healthy volunteers and lung cancer patients with anti-PD-1 therapy. Furthermore, *p53^fl/fl^LSL-Kras^G12D^*, *Ccr7^-/-^*, *Cd93^-/-^* mice and CD11c-DTR mice were generated. Specifically, EM, NTA and western blotting were utilized to identify Tumor extracellular vesicles (TEVs). EV labeling, detection of EV uptake in vitro and in vivo, degradation of EV proteins and RNAs were performed to detect the role of TEVs in tumor progression. Pleural mesothelial cells (pMCs) were isolated to investigate related signaling pathways. Recombinant proteins and antibodies were generated to test which antibody was the most effective one to increase CCL21a in p-pMCs. RNA-Seq, MiRNA array, luciferase reporter assay, endothelial tube formation assay, protein labeling and detection, transfection of siRNAs and the miRNA mimic and inhibitor, chemotaxis assay, immunohistochemical staining, flow cytometry, Real-time PCR, and ELISA experiments were performed.

**Results:** We show that CD93 of pMCs reduced lung tumor migration of dendritic cells by preventing pMCs from secreting CCL21, thereby suppressing systemic anti-lung tumor T-cell responses. TEV-derived miR-5110 promotes CCL21 secretion by downregulating pMC CD93, whereas C1q, increasing in tumor individuals, suppresses CD93-mediated CCL21 secretion. CD93-blocking antibodies (anti-CD93) inhibit lung tumor growth better than VEGF receptor-blocking antibodies because anti-CD93 inhibit tumor angiogenesis and promote CCL21 secretion from pMCs. Anti-CD93 also overcome lung tumor resistance to anti-PD-1 therapy. Furthermore, lung cancer patients with higher serum EV-derived miR-5193 (human miR-5110 homolog) are more sensitive to anti-PD-1 therapy, while patients with higher serum C1q are less sensitive, consistent with their regulatory functions on CD93.

**Conclusions:** Our study identifies a crucial role of CD93 in controlling anti-lung tumor immunity and suggests a promising approach for lung tumor therapy.

## Introduction

Lung cancer has the highest mortality and second highest incidence, accounting for 18% of deaths from malignant tumors 1. Patients with lung tumors have benefited from molecular-targeted therapies. However, eventual resistance to molecular-targeted therapies is inevitable 2. Immune checkpoint blockade, such as anti-programmed death-1 (PD-1) and anti-PD-ligand 1 antibodies, has shown clinical efficacy in advanced lung cancer. However, the therapeutic response rate is only 14-20% due to therapy resistance 3, 4. Therefore, the treatment of lung tumors is still a substantial challenge.

The pleura, comprising a monolayer of mesothelial cells (MCs), has diverse functions. MCs reduce friction between serosal surfaces by secreting phosphatidylcholine and participate in initiating and resolving serosal inflammation and repair 5. MCs are also believed to mediate tumor cell adhesion 6. Tumor-derived extracellular vesicles (TEVs) are responsible for tumor progression due to their critical roles in immunosuppression and metastasis. TEVs with high PD-L1 levels induce systemic immunosuppression and are critical to tumor progression 7. TEV integrins determine pre-metastatic niche formation, thereby leading to tumor organotropic metastasis 8. In contrast, TEVs are also demonstrated to participate in tumor suppression. HSP70-positive TEVs inhibit tumor progression by stimulating migratory and cytolytic activity of NK cells 9. TEVs potentially activate dendritic cells (DCs), thereby inhibiting murine hepatocellular carcinoma growth 10. In a previous study, TEVs were revealed to induce the apoptosis of peritoneal MCs, leading to peritoneal dissemination of tumor cells 11. Given the diverse target cells of TEVs, we hypothesized that TEVs might regulate antitumor immunity by altering the functions of pleural MCs (pMCs).

CD93 (C1qR_p_) is a C-type lectin-like type I transmembrane protein that plays a role in the phagocytosis of apoptotic cells and regulation of processes involved in innate immunity and inflammation 12, 13. CD93 has been shown to facilitate tumor progression by promoting angiogenesis 14. Blockade of CD93 normalizes tumor vasculature, thereby benefiting tumor therapy 15. However, whether CD93 has a direct role in regulating antitumor immunity remains to be explored.

Here, we demonstrate that intrapleurally injected TEVs notably inhibits lung tumor growth. Mechanistically, TEV-derived miR-5110 induces CCL21 secretion from pMCs by downregulating CD93, thereby promoting DC migration into lung tumors and subsequent activation of systemic anti-lung tumor immunity. Blockade of CD93 with monoclonal antibodies against CD93 (anti-CD93) markedly inhibited lung tumor growth by inducing CCL21 secretion from pMCs and preventing tumor angiogenesis, thus showing better efficacy than blocking vascular endothelial growth factor receptor (VEGFR). Furthermore, blocking CD93 reverses lung tumor resistance to anti-PD-1 therapy effectively. Therefore, we reveal the unique role of pMC CD93 in regulating systemic anti-lung tumor immunity and develop a potential therapeutic agent for lung tumors.

## Results

### Intrapleurally injected TEVs suppress lung tumor growth by promoting DC recruitment

**Figure 1:**
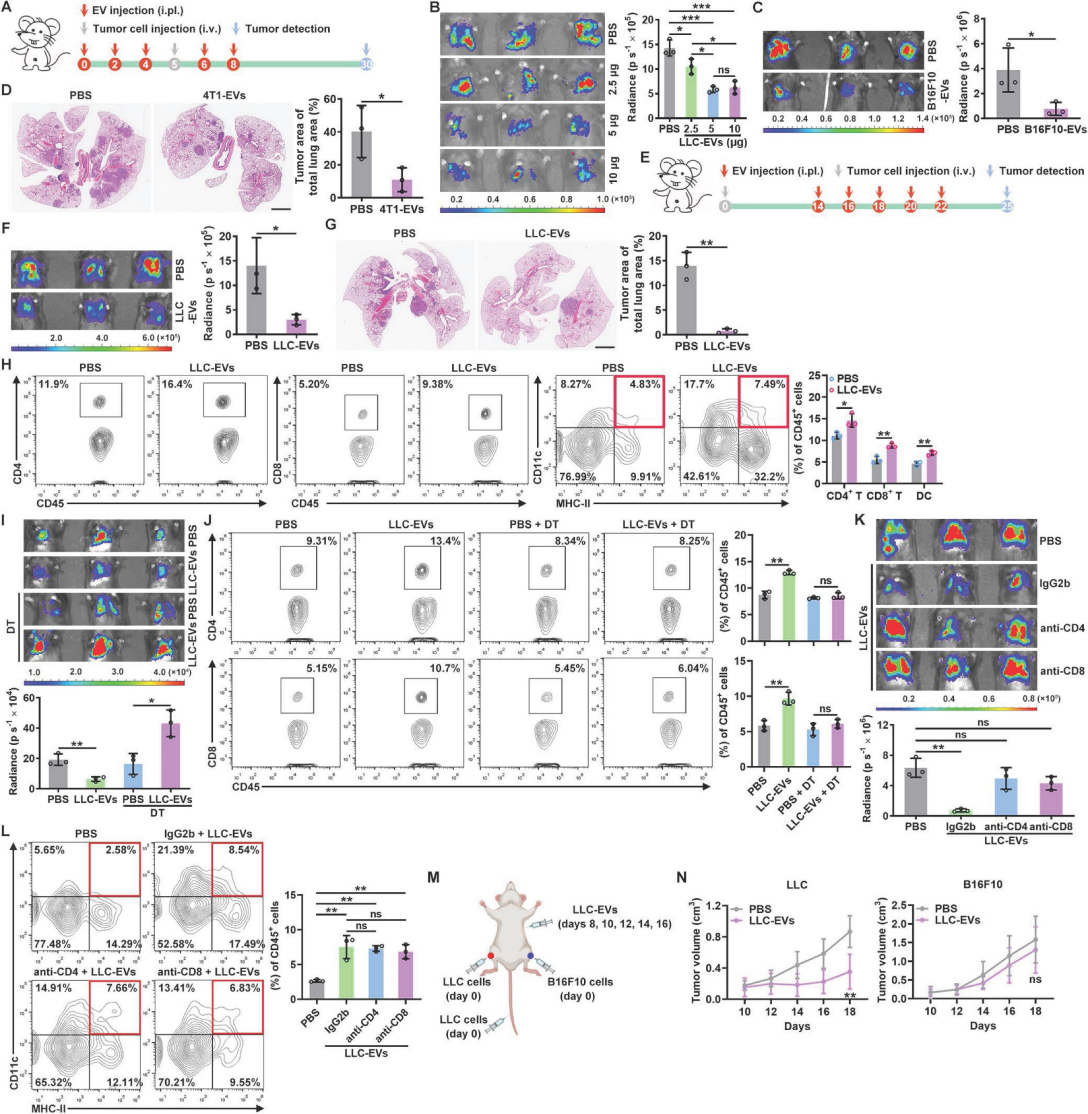
Intrapleurally injected TEVs suppress lung tumor growth by promoting DC recruitment. **A**, Schematic of the EV and tumor cell treatment protocol. **B**, **C**, The lung tumor size in mice injected with LLC-Luci cells (**B**) or B16F10-Luci cells (**C**) and the indicated doses of LLC-EVs (**B**) or 5 μg B16F10-EVs (**C**) as shown in **A** was evaluated with an *in vivo* imaging system (IVIS) on Day 30. **D**, 4T1 cells were subcutaneously transplanted into mice on Day 0, and the mice were intrapleurally injected with 5 μg 4T1-EVs on Days 26, 28, 30, 32 and 34. Lung tumors were detected and quantified by H&E staining on Day 37. **E**, Schematic of the EV and tumor cell treatment protocol. **F**, The lung tumor size in mice treated with LLC-Luci cells and 5 μg LLC-EVs, as shown in **E** was monitored with an IVIS on Day 25. **G**, Spontaneous lung tumors were induced in* p53^fl/fl^LSL-Kras^G12D^* mice by administration of adenoviruses expressing Cre recombinase via intranasal drip on Days 0 and 1. Then, the mice were intrapleurally injected with 5 μg LLC-EVs on Days 30, 32, 34, 36 and 38. Lung tumors were detected and quantified by H&E staining on Day 41. Scale bar, 2 mm. **H**, TILs from mice described in **e** were detected by flow cytometry. **I-L**, Mice were treated with LLC-Luci cells and 5 μg LLC-EVs as shown in **e**, along with DT (**I**,** J**) or IgG, an anti-CD4 antibody or an anti-CD8 antibody (**K**, **L**). The lung tumor size was monitored with an IVIS (**I**, **K**), and the frequencies of CD4^+^ and CD8^+^ T cells (**J**) or DCs (**L**) among TILs were determined by flow cytometry on Day 25. **M**, **N**, Mice received the indicated treatment (**M**), and the tumor size was then measured (**N**). Representative results from three independent experiments are shown (*n* = 3, except for *n* = 5 in **N**). *P < 0.05; **P < 0.01; ***P < 0.001; ns, not significant (unpaired two-tailed Student's* t* test except for one-way ANOVA followed by Turkey's test in **K**, **L**; mean and s.d.).

TEVs promote pre-metastatic niche formation, thereby accelerating tumor metastasis 16, 17. To characterize the role of TEVs in pleural metastasis of lung cancer, we intravenously injected luciferase-expressing murine LLC Lewis lung tumor (LLC-Luci) cells into mice that had previously been treated with EVs from LLC cells (LLC-EVs) (Figure S1A-C) by intrapleural injection (Figure 1A). Beyond expectation, we found that LLC-EVs dose-dependently inhibited the growth of LLC lung tumors (Figure 1B). However, neither intraperitoneal (i.p.) nor subcutaneous (s.c.) injection of LLC-EVs affected the development of LLC tumors. Intravenous (i.v.) injection of TEVs can promote lung pre-metastatic niche formation by activating alveolar epithelial TLR3 16. Consistent with this study, we found that i.v. injection of LLC-EVs notably promoted LLC tumors (Figure S1D). We also found that previous intrapleural injection of EVs from murine B16F10 melanoma cells (B16F10-EVs) inhibited the growth of lung metastatic B16F10-Luci tumors (Figure 1C). In addition, the previous intrapleural injection of B16F10-EVs or EVs from murine 4T1 breast cancer cells from mice on a BALB/c background (4T1-EVs) inhibited the growth of LLC tumors (Figure S1E-F), indicating a tumor antigen- and MHC-independent inhibition of lung tumor growth. To test the effect of intrapleurally injected TEVs on spontaneous lung metastatic tumors, we subcutaneously transplanted mice with 4T1 cells, which frequently metastasize to the lungs, and found that previous intrapleural injection with 4T1-EVs also inhibited the growth of 4T1 spontaneous lung metastatic tumors (Figure 1D). To determine whether intrapleural injection of TEVs has a therapeutic effect on lung tumors, LLC-EVs were administered after tumor implantation (Figure 1E). Intrapleural injection of LLC-EVs significantly inhibited the growth of preestablished LLC lung tumors (Figure 1F). In addition, when spontaneous lung tumors were induced in *p53^fl/fl^LSL-Kras^G12D^* mice, intrapleural injection of LLC-EVs also showed tumor suppressive effects (Figure 1G). These results demonstrate that intrapleural injection of TEVs results in substantial suppression of lung tumor growth.

To find out how intrapleural injection of TEVs suppresses lung tumor growth, we examined tumor-infiltrating leukocytes (TILs) in LLC lung tumors and found that intrapleural injection of LLC-EVs significantly increased the frequencies of CD4^+^ T cells, CD8^+^ T cells and DCs among TILs but did not alter the frequencies of macrophages, NK cells, neutrophils and B cells (Figure 1H and Figure S1G). We supposed that intrapleural injection of TEVs probably promotes DC recruitment into lung tumors and subsequent presentation of tumor antigens to CD4^+^ and CD8^+^ T cells, thereby expanding these populations. To test this, we first used CD11c-diphtheria toxin receptor (DTR) mice, in which DCs are selectively eliminated upon DT administration, and found that depletion of DCs reversed the growth trend of LLC lung tumors induced by intrapleural injection of LLC-EVs and eliminated the increases in CD4^+^ and CD8^+^ T cells among TILs (Figure 1I-J). Depletion of either CD4^+^ or CD8^+^ T cells abrogated the tumor suppression induced by intrapleural injection of LLC-EVs (Figure 1K). However, depletion of neither CD4^+^ nor CD8^+^ T cells affected the increase in DCs among TILs (Figure 1L), suggesting that the changes in the DC population preceded the changes in the CD4^+^ and CD8^+^ T cell populations.

To assess the effect of intrapleural injection of LLC-EVs on systematic antitumor immunity, we subcutaneously inoculated LLC or B16F10 tumors into each flank of mice bearing LLC lung tumors and treated these mice with intrapleural injection of LLC-EVs (Figure 1M). We found that the growth of LLC but not B16F10 tumors was significantly blunted by LLC-EVs (Figure 1N). However, in the absence of lung tumors, even if an increase in lung DCs was observed, intrapleural injection of LLC-EVs could not suppress the growth of LLC tumors (Figure S1H-I). Furthermore, intrapleural injection of LLC-EVs did not increase the frequencies of CD4^+^ and CD8^+^ T cells in the lungs (Figure S1H). Collectively, these results indicate that intrapleural injection of TEVs promotes the recruitment of DCs into lung tumors and the subsequent induction of tumor antigen-specific T-cell responses, which probably patrols whole body with circulation and exerts systemic antitumor effects.

### TEV-induced CCL21a secretion from pMCs promotes lung migration of DCs

**Figure 2:**
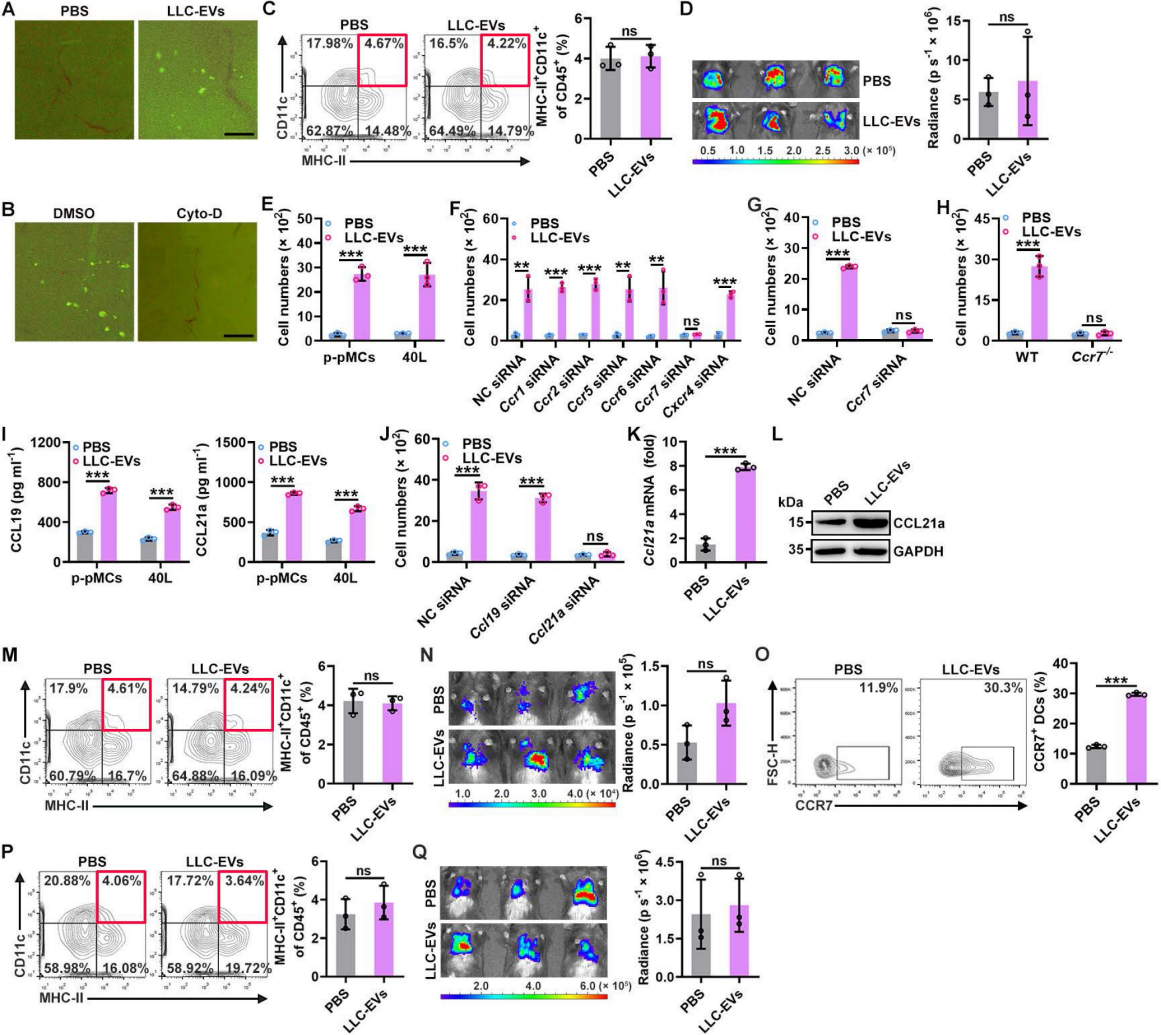
TEV-induced CCL21a secretion from pMCs promotes lung migration of DCs. **A**, Mice were intrapleurally injected with 20 μg CFSE-labeled LLC-EVs. After 24 h, incorporation of EVs into the pleura was detected by stereomicroscopy. **B-D**, Mice were treated with LLC-Luci cells and CFSE-labeled LLC-EVs as shown in Figure 1E, except that 0.25 mg kg^ -1^ Cyto-D was intrapleurally injected 2 h before each EV injection. The incorporation of EVs into the pleura was detected by stereomicroscopy (**B**), the DC frequency among TILs was determined by flow cytometry (**C**), and the lung tumor size was monitored with an IVIS (**D**). **E-J**, P-pMCs (**E**, **G-I**) and 40L cells (**F**, **J**) without mRNA silencing (**E**-**I**) or with the indicated siRNA silencing (**J**) were stimulated with 2.5 μg ml^-1^ LLC-EVs. After 24 h, chemotaxis of DCs without mRNA silencing (**E**, **I**,** J**), with the indicated siRNA (**F*,* G**) silencing, or chemotaxis of *Ccr7^-/-^* DCs (**H**) induced by supernatants from these cells was examined by a Transwell chemotaxis assay (**E-H**, **J**); the CCL19 and CCL21a levels in supernatants from these cells were measured by ELISA (**I**). (**K**, **L**) Mice were intrapleurally injected with 5 μg LLC-EVs. After 24 h, the pleural *Ccl21a* mRNA (**K**) and protein (**L**) levels were determined by real-time PCR (**K**) and western blotting (**L**). (**M-Q**) wild-type (WT) (**M-O**) and *Ccr7^-/-^* (**P**, **Q**) mice were treated with LLC-Luci cells and LLC-EVs as shown in Fig. 1e with (**M**, **N**) or without (**O-Q**) intrapleural injection of 10 μg cholesterol-conjugated *Ccl21a* siRNAs 24 h before each EV injection. The DC frequency among TILs (**M**,** P**) and the CCR7^+^ DC frequency (**O**) were analyzed by flow cytometry (**M**,** O**,** P**), and the lung tumor size was monitored with an IVIS (**N**,** Q**). Scale bar, 25 μm. Representative results from three independent experiments are shown (*n* = 3). ***P < 0.001; ns, not significant (unpaired two-tailed Student's* t* test; mean and s.d.).

To explore how intrapleural injection of TEVs promotes lung migration of DCs, we first compared the *in vivo* distribution of LLC-EVs administered by i.v. or intrapleural injection and found no apparent differences when determined with an IVIS (Figure S2A). However, intravenously injected LLC-EVs were mainly located in the lungs, while quite a few intrapleurally injected LLC-EVs were located at lung edges when visualized by fluorescence microscopy (Figure S2B). The pleural cavity is surrounded by the pleura, which is composed of a monolayer of pMCs. Therefore, pMCs probably took up most of the intrapleurally injected LLC-EVs. To confirm this hypothesis, we isolated primary pMCs (p-pMCs) and detected high MC marker mesothelin levels on isolated p-pMCs (Figure S2C). We also introduced murine 40L pleural mesothelioma cells 18. As expected, we found that both primary pMCs (p-pMCs) and 40L cells efficiently took up LLC-EVs *in vitro* (Figure S2D). LLC-EVs were also incorporated into the pleura (Figure 2A). Cytochalasin D (Cyto-D) is a potent inhibitor of actin polymerization, which can inhibit EV internalization by recipient cells. Intrapleural injection of Cyto-D before each LLC-EV injection inhibited the incorporation of LLC-EVs into the pleura, abrogated the increase in DCs in lung tumors and abolished tumor suppression (Figure 2B-D). However, intrapleural injection of Cyto-D after each LLC-EV injection did not prevent these effects (Figure S2E-G). These results indicate that TEV uptake by pMCs is required for lung tumor suppression mediated by intrapleural injection of TEVs.

Next, we investigated the effect of TEVs on pMCs. A previous study showed that TEVs induced the apoptosis of peritoneal MCs 11. However, we found that LLC-EVs slightly enhanced the viability of 40L cells at a concentration of 5 μg ml^-1^ (Figure S2H). Then, we examined whether TEVs affect the chemoattraction of bone marrow-derived DCs (BMDCs) by MCs and found that supernatants from either LLC-EV-stimulated p-pMCs or 40L cells had an enhanced ability to chemoattract BMDCs *in vitro* (Figure 2E). DCs are reported to express CCR1, CCR2, CCR5, CCR6, CCR7 and CXCR4 19; thus, we silenced these receptors individually (Figure S2I) and found that silencing of CCR7 but not the other receptors completely abolished the TEV-induced increase in the ability of 40L cell supernatants to chemoattract BMDCs (Figure 2F). Similarly, TEVs no longer promotes supernatants of p-pMCs to chemoattract CCR7-silenced or CCR7-deficient BMDCs (Figure 2G-H). CCL19 and CCL21a are the known ligands of CCR7 20. LLC-EVs notably increased the protein and mRNA levels of CCL19 and CCL21a in p-pMCs and 40L cells (Figure 2I and Figure S2J). However, knockdown (KD) of CCL21a but not CCL19 abolished the increase in the chemoattraction of BMDCs by supernatants from LLC-EV-stimulated 40L cells (Figure 2J and Figure S2K). Thus, TEVs promote the chemoattraction of BMDCs by pMCs *in vitro* by inducing CCL21a secretion.

Subsequently, we verified whether intrapleural injection of TEVs induces CCL21a secretion from pMCs *in vivo*. Intrapleural injection of LLC-EVs significantly increased the pleural mRNA and protein levels of CCL21a (Figure 2K-L). Therefore, we intrapleurally injected cholesterol-conjugated *Ccl21a* siRNA and confirmed the decrease in the pleural *Ccl21a* mRNA level (Figure S2L). Upon silencing of CCL21a in the pleura, intrapleural injection of LLC-EVs no longer increased the DC population in lung tumors or induced tumor suppression (Figure 2M-N). Then, we determined the role of CCR7 in DC recruitment induced by intrapleural injection of TEVs. First, we confirmed that intrapleural injection of LLC-EVs increased the frequency of CCR7^+^ DCs in lung tumors (Figure 2O). Then, we found that intrapleural injection of LLC-EVs did not increase the frequency of DCs in lung tumors or suppress tumor growth in *Ccr7^-/-^* mice (Figure 2P-Q). Collectively, these results indicate that intrapleural injection of TEVs induces CCL21a secretion from pMCs, which increases the recruitment of CCR7^+^ DCs into lung tumors.

### A decreased CD93 level of pMCs induces CCL21a secretion by TEVs

**Figure 3:**
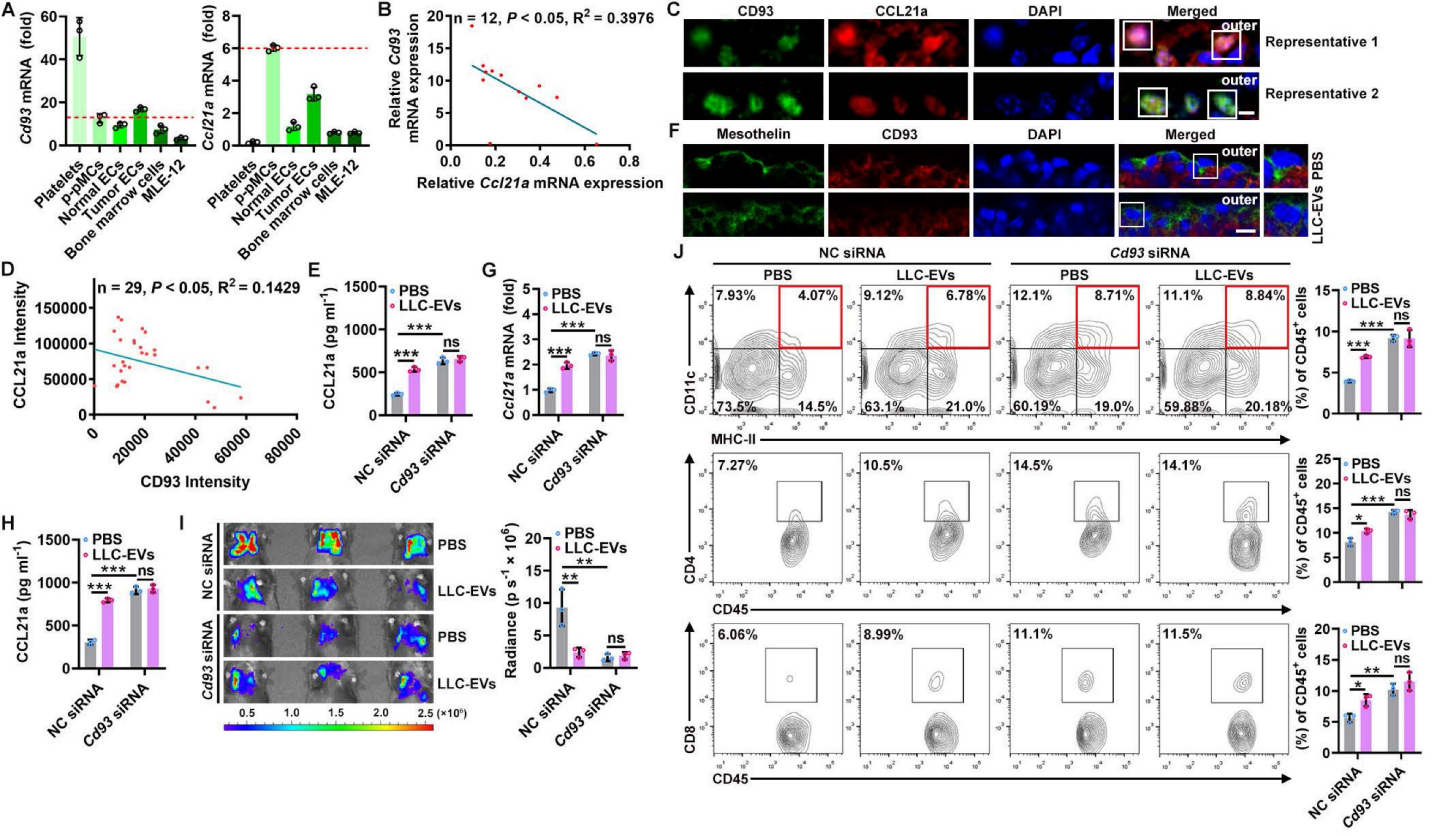
A decrease in CD93 level of pMCs induces CCL21a secretion by TEVs. **A**, Levels of *Cd93* and* Ccl21a* mRNA in the indicated cells were detected by real-time PCR.** B**, Correlation of pleural *Cd93* and* Ccl21a* mRNA levels in LLC lung tumor-bearing mice.** C**, **D**, CD93 and CCL21 proteins in pMCs of LLC lung tumor-bearing mice were detected by immunofluorescence microscopy (**C**) and the correlation of CD93 and CCL21 protein levels was analyzed (**D**). Scale bar, 5 μm. **E**, 40L cells with CD93 silencing were stimulated with 2.5 μg ml^-1^ LLC-EVs for 24 h. The CCL21a level in culture supernatants of these cells was measured by ELISA. **F**, Mice were intrapleurally injected with 20 μg LLC-EVs. Twenty-four hours later, CD93 in pMCs was detected by immunofluorescence staining. Scale bar, 10 μm. **G-J**, Mice were treated with LLC-Luci cells and LLC-EVs as shown in Figure 1E, with intrapleural injection of 10 μg cholesterol-conjugated *Cd93* siRNAs 24 h before each EV injection. Pleural *Ccl21* mRNA (**G**) and protein (**H**) levels were determined by real-time PCR (**G**) and ELISA (**H**) 24 h after the last EV injection. Lung tumor size was monitored with an IVIS (**I**), and DC, CD4^+^ T and CD8^+^ T frequency among TILs was determined by flow cytometry (**J**) on Day 25. Representative results from three independent experiments are shown (*n* = 3). *P < 0.05; **P < 0.01; ***P < 0.001; ns, not significant (unpaired two-tailed Student's* t* test in **A**; Spearman rank-order correlation test in **B**,** D**; one-way ANOVA followed by Turkey's test in **E**, **G-J**; mean and s.d.).

To explore how TEVs induce CCL21a secretion from pMCs, we analyzed 40L cells treated with or without LLC-EVs by RNA sequencing (RNA-Seq) and found that there were 109 differentially expressed genes (DEGs) in LLC-EV-treated 40L cells (Figure S3A). Then, we analyzed the correlation between *Ccl21a* and 31 DEGs with |Log_2_fold change (FC)| ≥ 2 (Figure S3B) and found that the expression of *Gm13054* (upregulated) and *Cd93* (downregulated) was highly correlated (correlation coefficient ≥ 0.90) with that of *Ccl21a* (Figure S3C). Moreover, the Log_2_FC in *Cd93* expression was much higher than that in *Gm13054* expression (Figure S3D). CD93 is preferentially expressed on platelets, endothelial cells (ECs), hematopoietic progenitors and alveolar epithelial cells 21. Except for platelets showing the highest *Cd93* mRNA expression, *Cd93* mRNA level in p-pMCs was comparable to that in ECs, bone marrow cells and MLE-12 mouse alveolar epithelial cells, even in tumor ECs reported to overexpress CD93, whereas *Ccl21a* mRNA was most highly expressed in p-pMCs (Figure 3A). The pleural *Cd93* but not *Ccl21a* mRNA level was also upregulated in tumor-bearing mice (Figure S3E). In addition, the *Cd93* and* Ccl21a* mRNA levels in tumor-bearing mice were inversely correlated (Figure 3B). Expression of CD93 and CCL21a proteins in pMCs were also detected by immunofluorescent staining, and CD93 and CCL21a protein levels were also inversely correlated (Figure 3C-D). In CD93-silenced 40L cells (Figure S3F), the CCL21a protein level was significantly increased. Additionally, LLC-EVs failed to induce CCL21a expression in these cells, suggesting that LLC-EVs induce CCL21a expression in a CD93-dependent manner (Figure 3E). Thus, these results indicate that CD93 negatively regulates CCL21a, which is specifically highly expressed in pMCs.

Then, we found that LLC-EVs also reduced CD93 protein expression in p-pMCs (Figure S3G) and that intrapleural injection of LLC-EVs reduced CD93 expression in situ when visualized by mesothelin (Figure 3F). LLC-EVs also CD93-dependently induced pleural CCL21a expression *in vivo*. Because, in mice with CD93 KD in situ by intrapleural injection of cholesterol-conjugated *Cd93* siRNA (Figure S3H), intrapleural injection of LLC-EVs no longer induced CCL21a mRNA and protein expression in the pleura (Figure 3G-H). In addition, lung tumor suppression could not be observed, along with the abolishment of increases in DCs, CD4^+^ T cells and CD8^+^ T cells (Figure 3I-J). More importantly, pleural CD93 silencing notably induced CCL21a secretion and antitumor immunity (Figure 3G-J). Thus, these results indicate that TEVs promote CCL21a secretion from pMCs by reducing CD93 expression.

### CD93 of pMCs is downregulated by TEV-derived miR-5110

**Figure 4:**
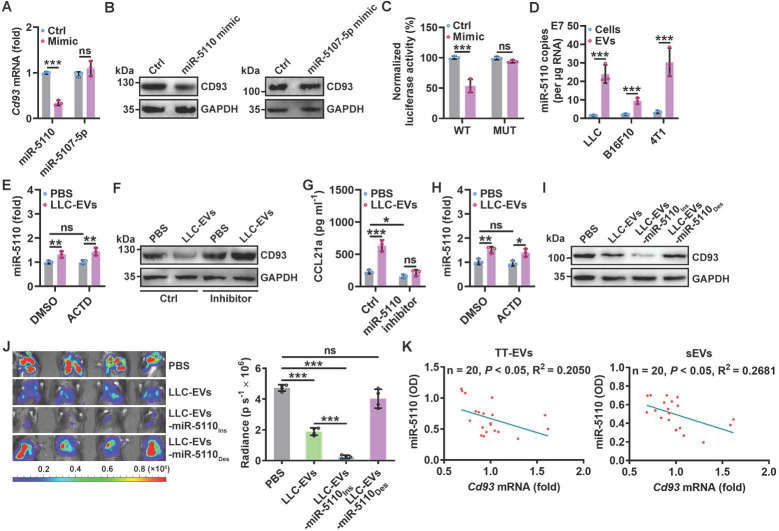
CD93 of pMCs is downregulated by TEV-derived miR-5110. **A**, **B**, *Cd93* mRNA (**A**) and CD93 protein (**B**) levels in 40L cells transfected with miR-5110 or miR-5107-5p mimics for 24 h were determined by real-time PCR (**A**) and western blotting (**B**). **C**, 40L cells were cotransfected with luciferase plasmids carrying the WT or MUT fragment of the *Cd93* 3'-UTR, *Renilla* luciferase plasmids, and the miR-5110 mimic. After 24 h, firefly luciferase activity was measured and normalized to *Renilla* luciferase activity. **D**, Absolute quantification of miR-5110 expression in the indicated cells and EVs by real-time PCR. **E**, 40L cells were stimulated with 2.5 μg ml^-1^ LLC-EVs for 8 h with or without 5 μg ml^-1^ ACTD. Then, miR-5110 in these cells was measured by real-time PCR. **F**, **G**, 40L cells transfected with the miR-5110 inhibitor were stimulated with 2.5 μg ml^-1^ LLC-EVs for 24 h. CD93 (**F**) and CCL21a (**G**) in these cells were detected by western blotting (**F**) and ELISA (**G**), respectively. **H**, Mice were intrapleurally injected LLC-EVs, along with or without 0.125 mg kg^-1^ ATCD injection 2 h ahead. miR-5110 in the pleura was measured by real-time PCR 8 h later. **I**, 40L cells were stimulated with 2.5 μg ml^-1^ LLC-EVs, LLC-EVs-miR-5110_Ins_ or LLC-EVs-miR-5110_Des_ for 24 h. CD93 in these cells was detected by western blotting. **J**, Mice were treated with LLC-Luci cells, and the indicated EVs, as shown in Figure 1E. Lung tumor size was monitored with an IVIS on Day 25. **K**, Correlation between miR-5110 levels in TT-EVs and sEVs with pleural *Cd93* mRNA level in LLC lung tumor-bearing mice. Representative results from two independent experiments are shown (*n* = 3). **P < 0.01; ***P < 0.001; ns, not significant (unpaired two-tailed Student's* t* test in **A**, **C**, **D**, **H**; Spearman rank-order correlation test in **K**; one-way ANOVA followed by Turkey's test in others; mean and s.d.).

Next, we investigated how CD93 in pMCs is downregulated by TEVs. First, we coincubated LLC-EVs with protease K with electroporation to digest total proteins of LLC-EVs (Figure S4A-B) and found that TEV proteins were not involved in the induction of CCL21a secretion by 40L cells (Figure S4C). Then, we coincubated LLC-EVs with RNase I in combination with electroporation to degrade RNAs (Figure S4D) and confirmed that these LLC-EVs no longer induced CCL21a secretion by 40L cells (Figure S4E), suggesting the decisive role of RNAs. MiRNAs enriched in exosomes are critical to exosome-mediated intercellular communication 22. To determine the miRNA(s) responsible for TEV-induced CCL21a secretion from pMCs, we analyzed the miRNAs in EVs from MLE-12 mouse lung epithelial type II cells (MLE-12-EVs), which did not inhibit CCL21a release from 40L cells (Figure S4F) and LLC-EVs by miRNA array. We found that there were 45 enriched miRNAs in LLC-EVs compared with MLE-12-EVs (Figure S4G). Then, the upstream miRNAs of *Cd93* were predicted with the miRDB and TargetScan databases, and 123 genes were identified from both databases (Figure S4H). After alignment of the enriched miRNAs and the predicted upstream miRNAs of *Cd93*, we found that miR-5110 and miR-5107-5p overlapped between these two groups of miRNAs (Figure S4I). Therefore, we examined the effect of these two miRNAs on CD93 expression. We found that miR-5110 but not miR-5107-5p mimic notably reduced the mRNA and protein levels of CD93 in 40L cells Figure 4A-B and Figure S4J). Then, we investigated whether CD93 is a direct target of miR-5110. TargetScan predicted two miR-5110 target sites with the same sequence in the *Cd93* 3'-UTR (Figure S4K). A luciferase reporter assay revealed that this sequence (WT) was a target of miR-5110, and mutated (MUT) sequence abolished the inhibition of luciferase activity by the miR-5110 mimic (Figure 4C and Figure S4K).

To test whether LLC-EV-derived miR-5110 downregulates CD93, we first confirmed that miR-5110 was significantly enriched in TEVs compared with the parental cells (Figure 4D). Furthermore, LLC-EV treatment significantly increased miR-5110 in 40L cells, which was not negated by transcription inhibitor Actinomycin D (ACTD), suggesting that TEV-mediated transfer rather than *de novo* synthesis of miR-5110 increases pMC miR-5110 (Figure 4E and Figure S4L). Then, we found that in 40L cells with miR-5110 KD, LLC-EVs no longer reduced CD93 and increased CCL21a, and that miR-5110 silencing alone caused an evident increased CD93 and decreased CCL21a (Figure 4F-G). Similar results were obtained by B16F10-EVs and 4T1-EVs (Figure S4M-O). Furthermore, intrapleural injection with LLC-EVs notably increased pleural miR-5110, even if pleura was pre-treated with ACTD (Figure 4H and Figure S4P). Subsequently, we obtained LLC-EVs with an increased miR-5110 level (LLC-EVs-miR-5110_Ins_) and LLC-EVs with a decreased miR-5110 level (LLC-EVs-miR-5110_Des_) from LLC cells transfected with the miR-5110 mimic or inhibitor, respectively (Figure S4Q). Compared with LLC-EVs, LLC-EVs-miR-5110_Ins_ and LLC-EVs-miR-5110_Des_ exhibited an increased and decreased ability, respectively, to downregulate CD93 in 40L cells (Figure 4I). Furthermore, LLC-EVs-miR-5110_Ins_ and LLC-EVs-miR-5110_Des_ exhibited improved and comparable abilities, respectively, to suppress LLC tumor growth (Figure 4J). Therefore, TEV-derived miR-5110 mediates CD93 downregulation in pMCs, leading to the inhibition of lung tumor growth. Subsequently, we found that miR-5110 levels in EVs from tumor tissues (TT-EVs) of LLC lung tumor-bearing mice and serum EVs (sEVs) were both inversely correlated with *Cd93* mRNA level in pMCs of these mice (Figure 4K). These results demonstrate that TEV-5110 level negatively indicates CD93 expression in pMCs.

### A decreased CD93 level of pMCs indicates increased T-cell responses in humans

**Figure 5:**
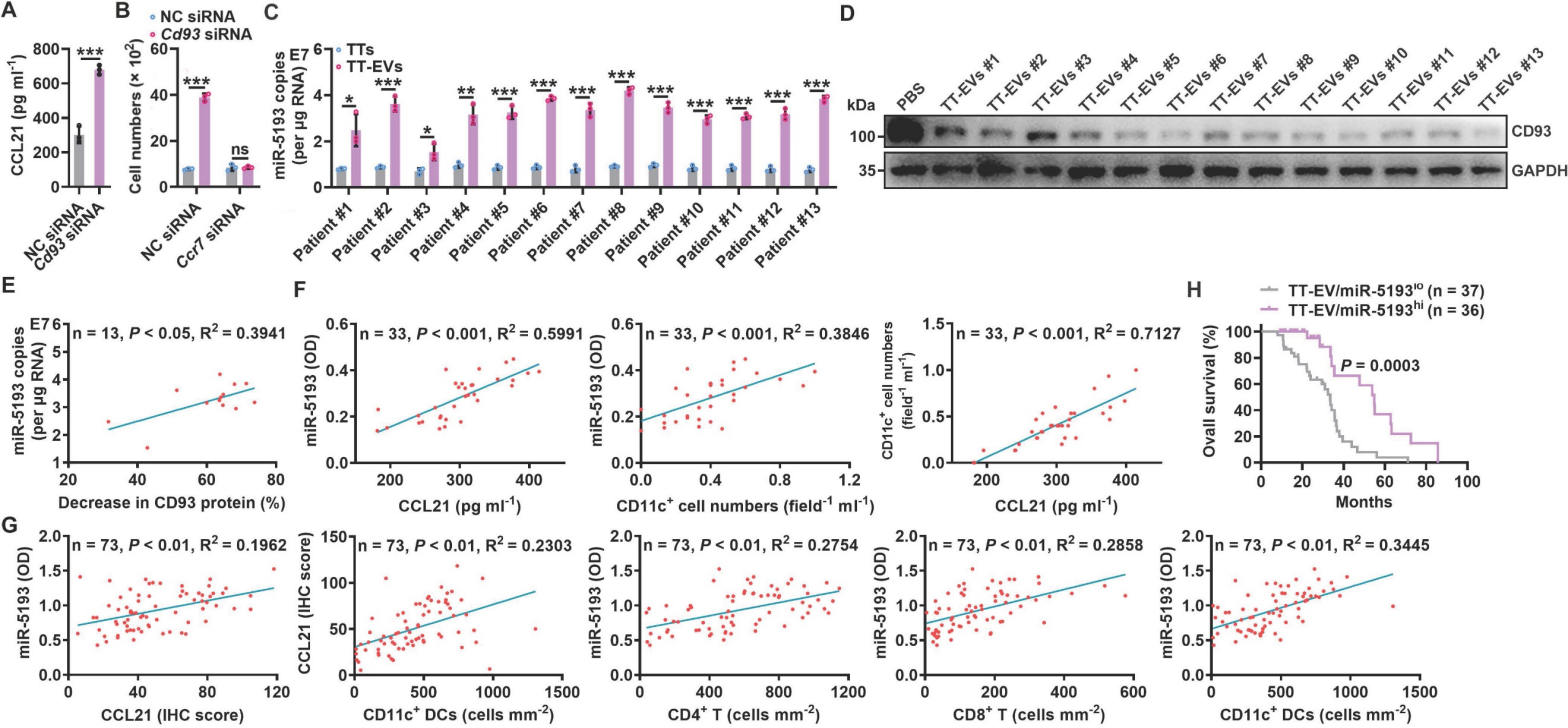
A decreased CD93 level of pMCs indicates increased T-cell responses in humans. **A**, CCL21 level in supernatants of NCI-H2452 cells with or without CD93 silencing was measured by ELISA. **B**, Chemotaxis of DCs with or without CCR7 silencing induced by supernatants of NCI-H2452 cells with or without CD93 silencing was evaluated by a Transwell chemotaxis assay. **C**, Absolute quantification of miR-5193 levels in TTs and TT-EVs by real-time PCR. **D**, NCI-H2452 cells were stimulated with 2.5 μg ml^-1^ TT-EVs for 24 h. The CD93 level in these cells was measured by western blotting. **E**, Correlation between TT-EV miR-5193 levels in **C** and inhibitory percentage of CD93 protein levels in **D**. **F**, **G**, Correlation between the indicated factors in MPE (**F**) and TTs (**G**). **H**, Overall survival curve of lung cancer patients with TT-EV/miR-5193^hi^ and TT-EV/miR-5193^lo^. Representative results from three independent experiments are shown (*n* = 3). *P < 0.05; **P < 0.01; ***P < 0.001; ns, not significant (unpaired two-tailed Student's* t* test in **A**-**C**; Spearman rank-order correlation test in **E-G**; log-rank test in** H**; mean and s.d.).

To elucidate whether CD93 of human MCs also regulates antitumor immunity in lung tumor patients, we first confirmed that NCI-H2452 human pleural mesothelioma cells also expressed higher levels of *Cd93* and *Ccl21* mRNA than human umbilical vein endothelial cells (HUVECs) (Figure S5A). Then, we found that silencing of CD93 in NCI-H2452 cells notably promoted CCL21 secretion and supernatant from these cells had increased ability to chemoattract human DCs, which was abolished when CCR7 in DCs was knocked down (Figure 5A-B and Figure S5B-C). In addition, we found that miR-5193 (human miR-5110 homolog) was enriched in TT-EVs from lung cancer patients, and TT-EVs significantly inhibited CD93 expression in NCI-H2452 cells (Figure 5C-D). Notably, miR-5193 level in TT-EVs was positively correlated with TT-EV ability to inhibit CD93 expression (Figure 5E), which suggests that TT-EV-derived miR-5193 is also a negative indicator for CD93 level in human pMCs.

We then evaluated the relationship between CD93 level in pMCs and T-cell responses in lung cancer patients. Since MCs of lung cancer patients were unavailable, we measured miR-5193 level in EVs instead of directly detecting CD93 level in pMCs to assess that. First, we determined the concentrations of CCL21, CD11c^+^ DCs and EV miR-5193 in malignant pleural effusions (MPEs) from lung cancer patients (Figure S5D and Table S1) and found that the EV miR-5193 concentration was positively correlated with the CCL21 and DC concentrations. Moreover, the CCL21 and DC concentrations were positively correlated (Figure 5F). Then, we also measured miR-5193 level in TT-EVs from 73 lung cancer patients (Table S2) and found that the TT-EV miR-5193 level was positively correlated with the CCL21 level and the numbers of CD11c^+^ DCs, CD4^+^ T cells and CD8^+^ T cells in tumor tissues and that the CCL21 level and DC number were also positively correlated (Figure 5G and Figure S5E). In addition, patients with high TT-EV miR-5193 levels (TT-EV/miR-5193^hi^: indicating low CD93 in pMCs) had better overall survival than patients with low TT-EV miR-5193 levels (TT-EV/miR-5193^lo^: indicating high CD93 in pMCs) (Figure 5H). Thus, CD93 in pMCs is also probably involved in the regulation of T-cell responses in lung cancer patients by promoting CCL21-mediated DC recruitment.

### Anti-CD93 suppress lung tumor growth by promoting CCL21 secretion from pMCs

**Figure 6:**
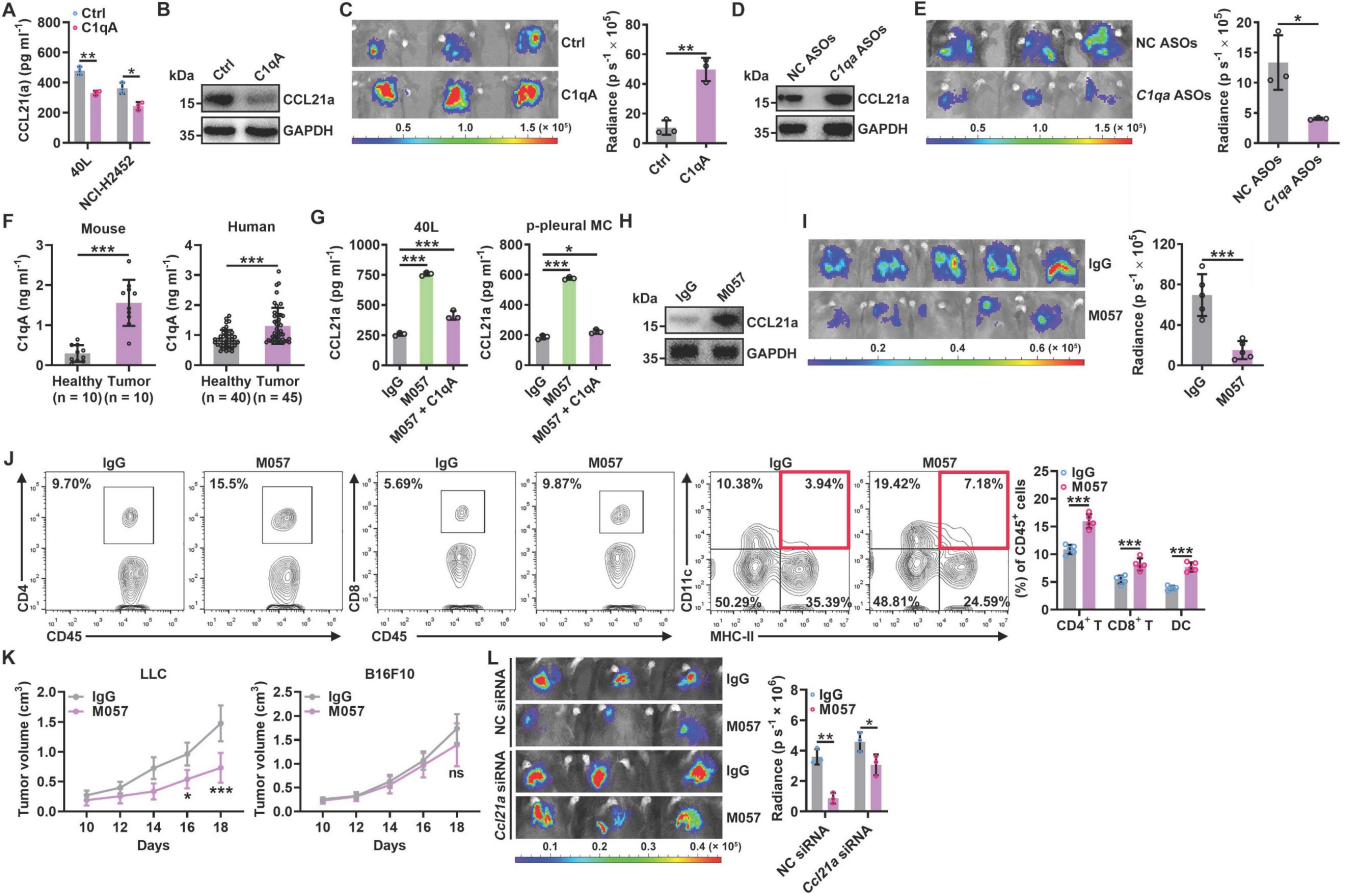
Anti-CD93 suppress lung tumor growth by promoting CCL21 secretion from pMCs. **A**, 40L or NCI-H2452 cells were stimulated with 2 μg ml^-1^ recombinant C1qA for 24 h. CCL21a or CCL21 level in supernatants of these cells was measured by ELISA. **B**, **C**, LLC-Luci tumor-bearing mice received intrapleural injection with 5 μg C1qA on Days 14, 16, 18, 20 and 22. Pleural CCL21a protein level was determined by western blotting (**B**), and lung tumor size was monitored with an IVIS (**C**) on Day 25. **D**, **E**, LLC-Luci tumor-bearing mice received i.v. injection with 100 μg cholesterol-conjugated* C1qa* ASOs on Days 14, 16, 18, 20 and 22. C1q in the liver was determined by western blotting (**D**), and lung tumor size was monitored with an IVIS (**E**) on Day 25. **F**, Serum C1qA levels in LLC lung tumor-bearing mice and lung tumor patients were measured by ELISA. **G**, 40L cells and p-pMCs were treated with 10 μg ml^-1^ M057 in the presence of 2 μg ml^-1^ C1qA for 24 h. CCL21a level in supernatants of these cells was measured by ELISA.** H-J**, LLC-Luci tumor-bearing mice were intravenously injected with 100 μg M057 on Days 14, 16, 18, 20 and 22. Pleural CCL21a protein level was determined by western blotting (**H**), lung tumor size was monitored with an IVIS (**I**) and the DC, CD4^+^ T cell and CD8^+^ T cell frequency among TILs analyzed by flow cytometry (**J**) on Day 25. **K**, LLC-Luci lung tumor-bearing mice were subcutaneously inoculated with LLC or B16F10 tumors on each flank, followed by treatment with 100 μg M057 on Days 8, 10, 12, 14 and 16. The tumor size was then measured.** L**, LLC-Luci lung tumor-bearing mice with or without pleural knockdown of CCL21a were intravenously injected with 100 μg M057 on Days 14, 16, 18, 20 and 22. Lung tumor size was monitored with an IVIS on Day 25. The day mice received tumor cells injected was regarded as Day 0. Representative results from two independent experiments are shown (*n* = 3, except for *n* = 5 in **i**, **k**). *P < 0.05; **P < 0.01; ***P < 0.001; ns, not significant (unpaired two-tailed Student's* t* test, except for one-way ANOVA followed by Turkeys' test in **g**; mean and s.d.).

Since pMC CD93 is critical to regulating anti-lung tumor immunity, we sought to determine the ligand(s) responsible for CD93 activation in pMCs. C1q, multimerin-2 (MMRN2) and insulin-like growth factor binding protein 7 (IGFBP7) are the known ligands of CD93 23. Overexpression of C1qA rather than MMRN2 or IGFBP7 significantly inhibited *Ccl21a* mRNA levels in 40L or NCI-H2452 cells (Figure S6A-B). In addition, recombinant C1qA stimulation significantly reduced CCL21a or CCL21 secretion from 40L or NCI-H2452 cells, respectively (Figure 6A). Intrapleural injection of recombinant C1qA also inhibited pleural CCL21a proteins and promoted lung tumor growth (Figure 6B-C). C1q is mainly synthesized in the liver, and cholesterol-conjugated oligonucleotides have excellent liver targeting 24. When C1qA in the liver was knocked down by cholesterol-conjugated *C1qa* antisense oligonucleotide (*C1qa* ASOs) (Figure S6C), we found a notable increase in pleural CCl21a expression and delayed lung tumor growth (Figure 6D-E). In addition, we detected greatly enhanced serum C1qA levels in lung tumor-bearing mice and lung tumor patients (Figure 6F). CD93 contains one C-type lectin domain (CTLD), one Sushi, five epidermal growth factor (EGF)-like domain and one Mucin domain 15, 25. Then, we constructed CD93 truncations with the indicated domains deleted (Figure S6D) and found that overexpression of CTLD, comparable to CD93-WT, was sufficient to suppress *Ccl21a* mRNA expression in 40L cells (Figure S6E-F). In addition, overexpression of CTLD rather than CD93 with CTLD deletion (CD93ΔCTLD) significantly inhibited CCL21a release from 40L cells, and the inhibitory effect was similar to CD93-WT (Figure S6G). These results suggest that CTLD of CD93 is involved in C1q-induced inhibition of CCL21. In conclusion, C1q is the ligand of CD93 responsible for the regulation of CCL21 in pMCs.

Then, we inferred that therapeutic effects of lung tumors could be obtained if C1q-mediated CD93 activation in pMCs is abolished by anti-CD93. Therefore, we generated a rabbit monoclonal antibody against mouse CD93 in which the constant region was replaced with mouse IgG (clone M057). The dissociation constant (*K_d_*) of M057 was 0.24 nM (Figure S7A). M057 stained bone marrow cells from WT mice but not *Cd93^-/-^* mice (Figure S7B). Moreover, a strong signal could be detected in the pleura of WT but not *Cd93^-/-^* mice received intravenous injection with Alexa Fluor 680-labeled M057 (Figure S7C). These results indicate M057 specifically bind to CD93.

When validated the functions of M057, we found that M057 treatment significantly improved CCL21a secretion by 40L cells and p-pMCs even in the presence of recombinant C1q (Figure 6G). In addition, M057 could not enhance CCL21a secretion by 40L cells with CD93 silencing, while reexpression of CD93 in these cells rescued the increase in CCl21a secretion induced by M057 (Figure S7D-E). Then, we administrated LLC lung tumor-bearing by serial doses of M057. We found that M057 notably increased pleural *Ccl21a* mRNA level in a dose-dependent manner, and 100 μg M057 was the optimal dose (Figure S7F). At the optimal dose of M057, pleural CCL21a protein level and LLC tumor growth were notably increased and suppressed, respectively (Figure 6H-I). Correspondingly, M057 significantly increased the frequencies of CD4^+^ T cells, CD8^+^ T cells and DCs among TILs (Figure 6J). In addition, M057 treatment also induced systemic tumor-specific immunity (Figure 6K). To elucidate whether M057 inhibits lung tumor growth by targeting CD93 in pMCs, we knocked down pleural CCL21a and found that M057 still showed antitumor efficacy in these mice. However, the antitumor effects were significantly blunted compared with mice without pleural CCL21a knockdown (Figure 6L). These results demonstrate that M057 suppresses lung tumor growth mainly by inhibiting pleural CCL21 secretion.

Next, we characterized the *in vivo* toxicity of M057 and found that M057 treatment did not increase the levels of alanine transaminase (ALT), aspartate aminotransferase (AST), bilirubin and creatinine in sera (Figure S7G). M057 treatment also did not cause apparent histopathological damage to the main organs, including the heart, liver, spleen, lungs and kidneys (Figure S7H). These results suggest the satisfying biosafety of M057.

### Anti-CD93 have better efficacy than anti-VEGFR against lung tumors

**Figure 7:**
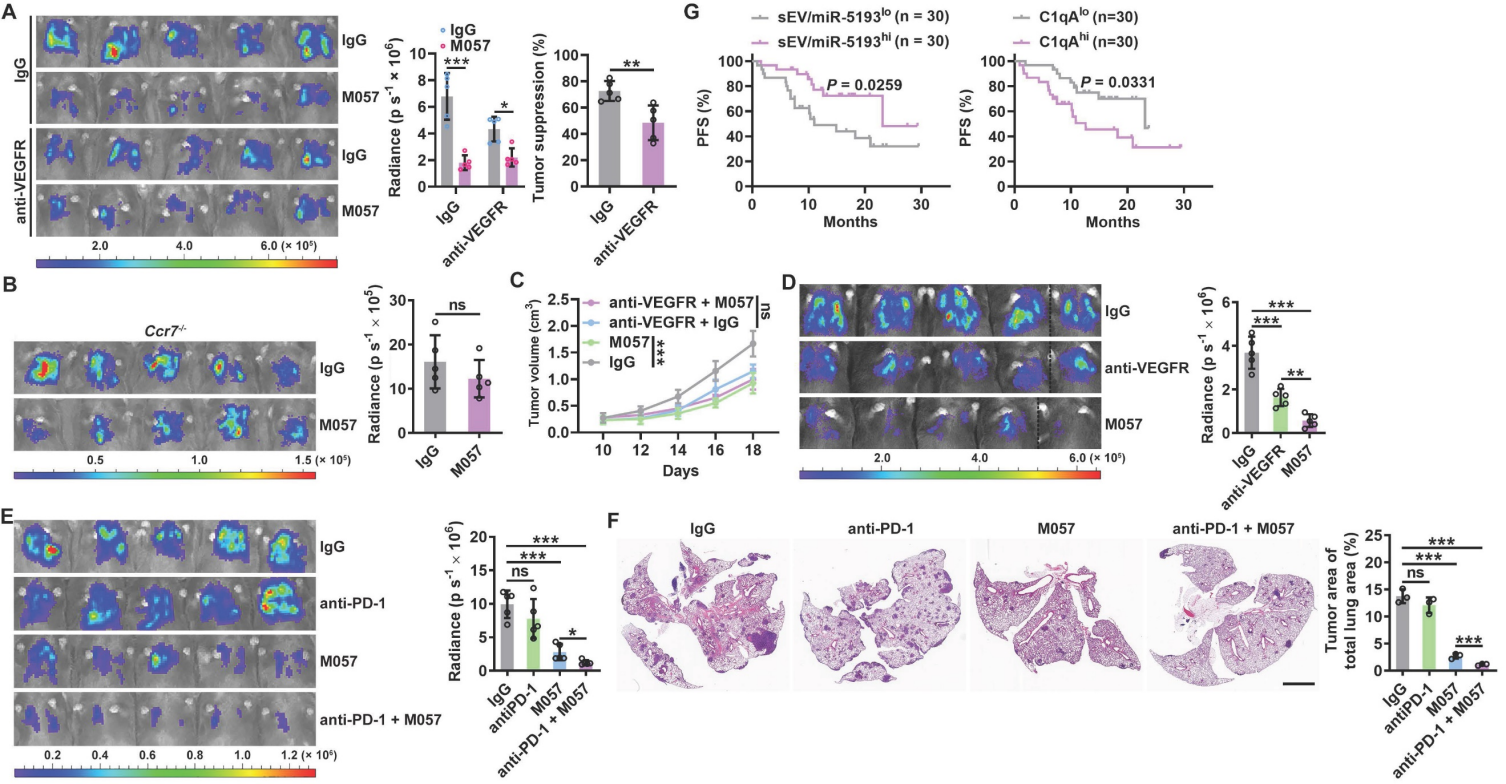
Anti-CD93 have better efficacy than anti-VEGFR against lung tumors. **A-C**, LLC-Luci lung tumor-bearing WT (**A**), *Ccr7^-/-^* (**B**) or LLC subcutaneous tumor-bearing WT (**C**) mice were intravenously injected with 40 μg anti-VEGFR on Days 13, 15, 17, 19 and 21 (**A**,** B**) or 7, 9, 11, 13 and 15 (**C**). Twenty-four h after every anti-VEGFR injection, the mice received intravenous injection with 100 μg M057 or mouse IgG. Lung tumor size was monitored with an IVIS 24 h on Day 25 (**A**, **B**), and subcutaneous tumor size was measured (**C**). **D**, LLC-Luci lung tumor-bearing mice were intravenously injected with 40 μg anti-VEGFR or 100 μg M057 on Days 14, 16, 18, 20 and 22. Lung tumor size was monitored with an IVIS on Day 25. **E**, **F**, B16F10 and 4T1 lung tumor-bearing mice were intravenously injected with 50 μg anti-PD-1 with or without 100 μg M057 on Days 14, 16, 18, 20 and 22. Lung tumor sizes were monitored with an IVIS (**E**) and detected by H&E staining (**F**) on Day 25. **G**, PFS of anti-PD-1-treated lung cancer patients relative to sEV/miR-5193 and serum C1qA. The day mice received tumor cells injected was regarded as Day 0. Scale bar, 2mm. Representative results from two independent experiments are shown (*n* = 5, except for *n* = 3 in **F**). *P < 0.05; **P < 0.01; ***P < 0.001; ns, not significant (unpaired two-tailed Student's* t* test in **A**-C; one-way ANOVA followed by Turkeys' test in **D-F**; log-rank test in** G**; mean and s.d.).

IGFBP7 and CD93 interaction blockade by anti-CD93 has been reported to suppress tumor growth by normalizing tumor vasculature 15. Given that the CTLD domain is required for both functions of CD93 to promote tumor angiogenesis and induce CCL21 secretion from MCs and that pleural silencing of CCL21 did not altogether abolish the antitumor effects of M057 (Figure 6L), we assumed that M057 might also show anti-lung tumor efficacy by normalizing tumor vasculature. First, we found that M057 also inhibited IGFBP7-induced EC angiogenesis *in vitro* (Figure S8A). Furthermore, although M057 treatment did not alter CD31^+^ vessel density in LLC lung tumor-bearing mice, the percentage of smooth muscle cell (indicated by α-smooth muscle actin, αSMA)- and pericyte (indicated by neural/glial antigen 2, NG2)-covered blood vessels in tumor tissues were notably increased (Figure S8B). In contrast, when M057-treated mice were intravenously injected with FITC-dextran, we found that the leakage area (dextran positive) of tumors was markedly reduced (Figure S8C). Although CD93 is downstream of VEGFR signaling 15, blockade of VEGFR by optimal therapeutic dose of anti-VEGFR (Figure S8D) blunted rather than abolished the antitumor effects of M057 in LLC lung tumor-bearing mice (Figure 7A). However, in LLC lung tumor-bearing *Ccr7^-/-^* mice with VEGFR blockade, M057 treatment no longer inhibited tumor growth (Figure 7B). In addition, in subcutaneous LLC tumor-bearing WT mice with VEGFR blockade, M057 treatment did not inhibit tumor growth as well (Figure 7C). These results suggest that M057 specifically inhibits lung tumor growth by not only promoting CCL21-mediated DC migration but also normalizing tumor vasculature.

Anti-CD93 and anti-VEGFR suppress subcutaneous tumor growth by normalizing tumor vasculature, thereby having similar antitumor effects 15. However, M057 normalizes tumor vasculature in lung tumors and promotes CCL21-mediated DC migration into tumors. Therefore, the therapeutic effects of M057 on lung tumors should be more robust than anti-VEGFR. As expected, we did observe that M057 inhibited lung tumor growth more pronouncedly than anti-VEGFR (Figure 7D). However, in *Ccr7^-/-^* mice, M057 and anti-VEGFR showed a comparable ability to inhibit lung tumor growth (Figure S8E), supporting that increased tumor infiltration of CCR7^+^ DCs contributes to the enhanced anti-lung tumor effects of M057.

“Cold tumors” with few or absent T cells resist anti-PD-1 therapy 26, 27. M057-mediated normalization of tumor vasculature and enhanced DC migration increase T cells in tumors. Therefore, M057 treatment probably reverses the resistance of “cold tumors” to anti-PD-1 therapy. As expected, B16F10 and 4T1 lung tumors that primarily resisted anti-PD-1 therapy were both significantly inhibited by M057 treatment (Figure 7E-F). More importantly, as a negative or positive regulator of CD93, high sEV-derived miRNA-5193 or C1qA were associated with favorable or unfavorable progression-free survival (PFS) after anti-PD-1 therapy in lung cancer patients (Figure 7G and Table S3).

## Discussion

In this study, we found that intrapleurally injected murine LLC-EVs and B16F10-EVs were mainly taken up by pMCs. These EVs were enriched in miR-5110, which transferred miR-5110 into pMCs, leading to CD93 downregulation, thus promoting CCL21 secretion from pMCs and subsequent lung tumor infiltration of DCs and activation of antitumor T cell responses. This mechanism is also mirrored in human lung cancer patients. When determining the concentrations of CCL21, CD11c^+^ DCs and EV miR-5193 in MPEs from lung cancer patients, we found that the EV miR-5193 concentration was positively correlated with the CCL21 and DC concentrations and the CCL21 and DC concentrations were positively correlated. Besides, TT-EV miR-5193 level was also positively correlated with the CCL21 level and the numbers of CD11c^+^ DCs, CD4^+^ T cells and CD8^+^ T cells in tumor tissues and the CCL21 level and DC number were also positively correlated. However, we did not measure the CD93 expression on patient pMCs in this study. Therefore, the correlations of CD93 on patient pMCs and the factors mentioned above are still not directly elucidated.

B16F10-EVs reportedly inhibited antitumor immunity by activating PD-1 signaling in T cells via PD-L1 28. LLC-EVs and B16F10-EVs are involved in tumor lung metastasis by transferring RNAs into lung epithelial cells 16. Unlike these studies, we demonstrated that intrapleurally injected LLC-EVs and B16F10-EVs exerted antitumor effects. As previously mentioned, TEVs comprise various components responsible for tumorigenesis or suppression 29. Together, the gambling of these two forces determines the TEV effects on tumor progression. According to our results, the primary recipient cells for TEVs are crucial to the outcome of this gambling. In addition, the tumor-genetic or tumor-suppressive effects of TEVs mask each other in tumor patients. Therefore, further elucidation of the complicated effects of TEVs on tumor progression is beneficial for understanding tumor pathogenesis and revealing novel targets for tumor therapy.

TEV-derived miRNAs have potential roles as biomarkers in tumor diagnosis 30. Our results showed that miR-5110 was significantly enriched in TEVs compared with the parental cells. We also found that miR-5193 was enriched in EVs from lung tumor tissues, suggesting the potential of miR-5193 as a diagnostic biomarker for tumors. However, the mechanism by which miR-5193 is enriched in TEVs and whether this enrichment is a lung tumor-specific or general tumor phenomenon is unclear. RNA-binding proteins control the sorting of miRNAs into EVs by recognizing specific motifs 31, 32. Therefore, it is possible that some RNA-binding proteins mediate the sorting of miR-5193 into TEVs and that this protein likely upregulates under tumor conditions.

In tumor-bearing mice, we found that the *Cd93* mRNA level was notably upregulated in pMCs. However, the expression of *Ccl21a* mRNA remained unchanged. Probably, tumor products stimulate *Ccl21a* mRNA expression in pMCs, which is inhibited by the upregulated CD93 signaling, leading to similar *Ccl21a* mRNA level observed under tumor conditions. Therefore, it can be reasonably inferred that inhibiting CD93 signaling in pMCs will potentially upregulate CCL21a in pMCs, activating anti-lung tumor immunity. In addition, although a high *Cd93* mRNA level was detected in platelets, pMCs, ECs and bone marrow cells, *Ccl21a* mRNA was especially highly expressed in pMCs. These results also suggest that enhancing CCL21a by inhibiting CD93 signaling in pMCs is precise for stimulating anti-lung tumor immunity.

In addition to promoting CD93 expression in pMCs, the tumor also activates the CD93 pathway in pMCs because we found that C1q was responsible for CD93-mediated inhibition of CCL21 production by pMCs, and the C1q level was enhanced in both mice with lung tumors and lung tumor patients. Knockdown of C1q in the liver significantly increased CCL21 level in the pleura and prevented lung tumor growth. Therefore, blocking CD93 should greatly benefit the activation of anti-lung tumor immunity. As expected, we did observe remarkably delayed growth of lung tumors in mice treated with M057. Furthermore, the CTLD domain is indispensable for CD93 function in suppressing CCL21, and CTLD is also required for CD93 function in promoting angiogenesis. Our results showed that M057 stimulated anti-lung tumor immunity via CCL21 and normalized tumor vasculature, thus exhibiting more robust efficacy than anti-VEGFR. VEGF inhibitors in tumor therapy often cause hypertension and nephrotoxicity 33, 34, which probably will not or less occur with M057 therapy, as CD93 is downstream of VEGF signaling. We also did not detect apparent toxicity in M057-treated mice. In addition, no noticeable abnormalities were detected in the maturation and distribution of bone marrow-derived cells in *Cd93^-/-^* mice 12, which further supports the safety of M057 therapy. In conclusion, M057 is an excellent alternative to VEGF inhibitors in lung tumor therapy.

“Cold” tumors in which pre-existing T-cell infiltration is scarce or absent are resistant to anti-PD-1 therapy 26, 27, such as B16F10 and 4T1 35. Blocking CD93 normalized tumor vasculature and promoted lung tumor infiltration of DCs. Normalized vasculature will also benefit tumor infiltration of T cells. Then, CD93 blocking-induced increased DCs can activate the infiltrated T cells, thus probably improving the “desertification” of T cells in “cold” tumors and enhancing responses to anti-PD-1 therapy. As expected, M057 effectively reversed resistance to anti-PD-1 therapy of B16F10 and 4T1 lung tumors. Furthermore, as a negative or positive regulator of CD93, high serum EV-derived miRNA-5193 or C1qA were associated with favorable or unfavorable PFS after anti-PD-1 therapy in lung cancer patients, which suggests that CD93 is also relative to responses to anti-PD-1 therapy in lung tumor patients and anti-CD93 probably efficiently enhance clinical responses to anti-PD-1 treatment in lung tumor patients. Collectively, anti-CD93 hold high potential in the treatment of lung tumor patients.

## Materials and Methods

### Human samples

Surgical specimens of Lung tumor tissues from 2010 to 2020 were frozen at -80 °C and preserved in the Biobank of Zhejiang Cancer Hospital. MPEs from lung cancer patients and blood from healthy volunteers and lung cancer patients with anti-PD-1 therapy were prospectively obtained from the Second Affiliated Hospital, Zhejiang University School of Medicine, and approved by the Ethics Committee. All patients and healthy volunteers were informed of the use of their samples, and signed consent forms were obtained.

For frozen surgical lung tumor samples, equal quality of tissues were cut and digested with 2 mg ml^-1^ collagenase type IV (Worthington Biochemical, Freehold, NK, USA) and 0.2 μg ml^-1^ DNase I (Sigma-Aldrich, St. Louis, MO, USA) in RPMI-1640 medium at 37 °C for 30 min on a shaker. The digestion was terminated by EV-depleted FBS and the supernatant was filtered through 0.22 μm filters (Millipore, Billerica, MA, USA) and used for centrifugation to isolate EVs. The remaining lung tumor tissues were fixed with formalin, embedded in paraffin, and ready for tissue microarrays and immunohistochemistry.

For MPE samples, equal volume of MPE was centrifuged. The cells in MPE were fixed with formalin, embedded in paraffin and ready for immunofluorescence staining. The supernatant of MPE was used to examine the expression of CCL21 by ELISA. The supernatant was also used for isolation of MPE-EVs.

For blood samples, the whole blood was centrifuged at 3500 × g for 10 min in the anticoagulant tube. The supernatant was used to test the expression of C1qA and EV miRNA levels by ELISA.

### Mice and cell lines

Male C57BL/6J, BALB/c and nude mice were (6-8 weeks old; 20 g average body weight) purchased from Joint Ventures Sipper BK Experimental Animal Co., Ltd. (Shanghai, China). *p53^fl/fl^LSL-Kras^G12D^* mice were kindly provided by Prof. Songming Ying (Zhejiang University, Hangzhou, China). *Ccr7^-/-^* mice were kindly provided by Prof. Jianhua Li (Fudan University, Shanghai, China). CD11c-DTR mice were purchased from The Jackson Laboratory (Farmington, CT, USA). *Cd93^-/-^* mice were kindly provided by Prof. Libo Su (Chinese Academy of Sciences, Beijing, China). Mice were housed under specific pathogen-free conditions, and the experimental protocol was reviewed and approved by the Animal Research Ethics Committee of Zhejiang University.

Murine 4T1 breast cancer and 3T3 fibroblast cells were purchased from the American Type Culture Collection (Manassas, VA, USA). Murine LLC-Luci lung cancer cells and B16F10-Luci melanoma cells were purchased from PerkinElmer (Shanghai, China). Murine 40L pMCs were provided by Dr. Binghao Li (Second Affiliated Hospital, Zhejiang University School of Medicine). Murine MLE-12 lung epithelial cells and HUVECs were provided by Prof. Yuehai Ke (Zhejiang University, Hangzhou, China). Human NCI-H2452 mesothelioma cells were purchased from Ningbo Mingzhou Biotechnology Co., Ltd. (Ningbo, Zhejiang, China). 4T1, LLC-Luci and NCI-H2452 cells were cultured in RPMI-1640 medium supplemented with 10% fetal bovine serum (FBS) (Thermo Fisher Scientific; Waltham, CA, USA). B16F10-Luci and 40L cells were cultured in DMEM supplemented with 10% FBS. MLE-12 cells were cultured in DMEF/F12 (v v^-1^ = 1 1^-1^) medium supplemented with 2% FBS. All cells were maintained in a humidified incubator at 37 °C with 5% CO_2_-95% air. All cell lines were routinely tested with a Mycoplasma Detection Kit (Lonza, Basel, Switzerland) and confirmed negative for mycoplasma contamination.

### Antibodies

Information on all antibodies used in this study is provided in Table S4.

### EV isolation

FBS was ultracentrifuged at 120,000 × g for 10 h to remove EVs and was then added to DMEM at a final concentration of 10% (v v^-1^). LLC, 4T1 and B16 cells were cultured in this medium to approximately 90% confluence in 10 cm cell culture dishes. Lung tumor tissues were cut and digested with 2 mg ml^-1^ collagenase type IV and 0.2 μmg ml^-1^ DNase I in RPMI-1640 medium at 37 °C for 30 min on a shaker. Digestion was terminated with EV-depleted FBS. Then, EVs were isolated as previously described 36. The culture medium, digested liquid and MPE were collected and centrifuged (4 °C) at 300 × g for 5 min to remove cells, 2,000 × g for 20 min to remove debris and apoptotic bodies, and 10,000 × g for 30 min to remove large EVs. After filtration through 0.22 μm filters, the supernatants were ultracentrifuged at 120,000 × g for 70 min at 4 °C. The final pellets were resuspended in ice-cold PBS. The protein contents in the EVs were quantified by a bicinchoninic acid protein assay (Thermo Fisher Scientific).

### EM

For negative EV staining, 200 mesh carbon films were hydrophilized with a glow discharge instrument at 15 mA for 25 s. The EV solution was pipetted onto 200 mesh carbon-coated copper grids and kept at room temperature (RT) for 1 min. After the excess suspension was removed with filter paper and the grid was washed twice with ddH_2_O, EVs were negatively stained with 2% uranyl acetate at RT for 1 min, and the excess suspension was removed and air dried. Images were acquired by EM (Tecnai G2 Spirit 120 kV, Thermo FEI, Hillsboro, OR, USA).

### NTA

To measure particle size and concentration, EVs were evaluated by NTA using a NanoSight NS300 system (Malvern PANalytical, Shanghai, China) configured with a 488 nm laser and high-sensitivity sCMOS camera; data were finally analyzed with NTA 3.3 software.

### Western blotting

For western blotting, total cells and EVs were washed with ice-cold PBS, lysed in SDS buffer on ice and boiled for 10 min at 100 °C. Then, proteins in the samples were separated by SDS-PAGE, transferred onto PVDF membranes (Millipore) and probed with the corresponding primary antibodies and horseradish peroxidase (HRP)-conjugated secondary antibodies. An ECL Kit (MultiSciences, Hangzhou, Zhejiang, China) was used to visualize the bands.

### Treatment of lung tumor-bearing mice

For intrapleural injection, mice were anesthetized with 1% pentobarbital sodium (Sigma-Aldrich), and 5 μg of LLC-EVs, B16F10-EVs or 4T1-EVs was injected into the pleural cavity in the left anterolateral thoracic area at the level of the xiphoid according to a previous description on Days 0, 2, 4, 6 and 8 37. Then, lung metastatic tumors were established by i.v. injection of 1 × 10^6^ LLC-Luci or B16F10-Luci cells on Day 5. To establish spontaneous lung metastatic tumors, 1 × 10^6^ 4T1 cells were subcutaneously transplanted into mice on Day 0, and the tumors were surgically excised on Day 16. Then, these mice were intrapleurally injected with 5 μg 4T1-EVs on Days 26, 28, 30, 32 and 34. To evaluate the therapeutic effect, mice were intravenously injected with 1 × 10^6^ LLC-Luci cells on Day 0 and then received intrapleural injection of 5 μg of LLC-EVs on Days 14, 16, 18, 20 and 22. In some experiments, these mice received intrapleural injections of 10 μg siRNAs or i.v. injection of 40 μg anti-VEGFR2 (Bio X Cell, West Lebanon, NH, USA) before each treatment. To induce spontaneous lung tumors, *p53^fl/fl^LSL-Kras^G12D^* mice received adenoviruses expressing Cre recombinase (2 × 10^6^ PFU ml^-1^) via intranasal drip on Days 0 and 1. Then, the mice were intrapleurally injected with LLC-EVs on Days 30, 32, 34, 36 and 38. To monitor the tumor burden, mice bearing luciferase-expressing tumors were anesthetized on Day 25 or 30 and intraperitoneally injected with 100 μg (per kg body weight) of luciferin (Promega, Beijing, China). Luminescence images were acquired with an IVIS (PerkinElmer, Waltham, MA, USA) after luciferin injection for 10 min. Photon flux in the total lung area was analyzed with Living Image software (PerkinElmer). Mice with spontaneous primary or metastatic lung tumors were sacrificed on Day 41 or 37, respectively. Then, the bilateral lung tissues were embedded in paraffin and stained with H&E. Images were acquired with an Olympus BX53 inverted microscope (Olympus, Tokyo, Japan). The metastatic lung tumor burden was calculated as the ratio of the total tumor area to the lung area.

### Isolation of TILs and flow cytometry

Lung tissues from lung metastasis models were dissected, washed and digested with 2 mg ml^-1^ collagenase type I (Worthington Biochemical), 2 mg ml^-1^ collagenase type IV (Worthington Biochemical), and 0.2 μg ml^-1^ DNase I (Sigma-Aldrich) in RPMI-1640 medium at 37 °C for 1 h on a shaker. The digestion process was terminated with RPMI-1640 medium containing 10% FBS. Then, the cell suspension was filtered through a 70 μm cell strainer, and RBCs were lysed.

For flow cytometry, a single-cell suspension was incubated first with FcR blocking antibody for 30 min at 4 °C and then with fluorophore-conjugated primary antibodies for 30 min at 4 °C for surface staining. Dead cells were excluded by staining with Fixable Viability Dye eFluor^TM^ (Thermo Fisher Scientific). Samples were injected into a Deflex flow cytometer (Beckman Coulter, Brea, CA, USA), and the data were analyzed with FlowJo software (Tree Star, Ashland, OR, USA).

### BMDC generation

BMDCs were generated as previously described 29. Briefly, BM mononuclear cells were prepared from suspensions of mouse tibias and femurs by depletion of red cells and cultured at a density of 2 × 10^6^ cells ml^-1^ in 6-well plates in RPMI 1640 medium supplemented with 10% FBS, 10 ng ml^-1^ recombinant murine GM-CSF and 1 ng ml^-1^ mouse IL-4. After 48 h of culture, nonadherent cells were gently removed by washing; the remaining loosely adherent clusters were cultured for another 48 h and harvested for subsequent experiments. To generate human DCs, peripheral blood mononuclear cells from healthy volunteers were isolated by density centrifugation of heparinized blood in Histopaque®-1077 (Sigma-Aldrich), resuspended in culture medium and allowed to adhere to 6-well plates. After incubation for 2 h at 37 °C, nonadherent cells were removed, and adherent cells were cultured in 3 ml of medium containing 10 ng ml^-1^ GM-CSF and 1 ng ml^-1^ IL-4. After 3 days, 1.5 ml of the medium was removed and replaced with the same fresh medium containing GM-CSF and IL-4. After 7 days of culture, DCs were harvested, washed, and used for subsequent experiments.

### Depletion of immune cell subsets and blockade of VEGFR

Mice were treated with LLC cells and LLC-EVs according to the protocol shown in Fig. 1e. To deplete DCs, CD11c-DTR mice received successive i.p. injections of 2 μg of DT every 2 days beginning on Day 11. To deplete CD4^+^ or CD8^+^ T cells, mice received successive i.p. injections of 60 μg anti-CD4 and 16 μg anti-CD8 antibodies (Bio X Cell) every 2 days beginning on Day 11. For the blockade of VEGFR, mice received successive i.v. injections of 40 μg of anti-VEGFR2 (Bio X Cell) every 2 days beginning on Day 13 or 14.

### EV labeling

EVs were labeled using VivoTrack 680 (Fluorescence, Beijing, China), PKH26 (Sigma-Aldrich) or CFSE (Thermo Fisher Scientific) according to the manufacturer's instructions. For VivoTrack 680 labeling, 150 μg of EVs in 200 μl of PBS were mixed with 42 μM VivoTrack 680 at RT for 30 min. For PKH26 labeling, 150 μg of EVs were resuspended in 100 μl of diluent C, and 0.4 μl of PKH26 ethanolic dye solution was added to another 100 μl of diluent C. Then, 100 μl of the EV suspension was mixed with the 100 μl of dye solution for another 5 min. For CFSE labeling, 150 μg of EVs in 200 μl of PBS was incubated with 7.5 μM CFSE at 37 °C for 30 min. Staining was stopped by adding an equal volume of exosome-depleted FBS (Thermo Fisher Scientific) and incubating for 1 min. Finally, all the unbound dye was removed by ultracentrifugation at 120,000 × g for 70 min, and the pellets were resuspended in 200 μl of PBS.

### Detection of EV uptake *in vitro* and *in vivo*

To detect the uptake of TEVs by MCs, p-pMCs and 40L cells were treated with 2.5 μg ml^-1^ CFSE-labeled LLC-EVs for 24 h. To detect the distribution of TEVs *in vivo*, 100 μg of VivoTrack 680-labeled LLC-EVs were intravenously or intrapleurally injected into mice. The mice were euthanized 24 h later, and the brain, heart, lungs, liver, spleen, kidneys and organs of the gastrointestinal tract were collected, and images were taken under an IVIS (PerkinElmer). To detect the distribution of TEVs in the lungs, 20 μg of PKH26-labeled LLC-EVs were intravenously or intrapleurally injected into mice. The lungs of these mice were collected 24 h later, embedded in Tissue-Tek™ Cryo-O.C.T. Compound (Thermo Fisher Scientific) and processed to obtain 10 μm sections. Nuclei were stained with 0.5 μg ml^-1^ DAPI for 20 min at RT. The cells and stained sections were observed by confocal microscopy (Olympus IX83-FV3000). To detect pleural TEV uptake, 20 μg of CFSE-labeled LLC-EVs were intrapleurally injected into mice. In some experiments, 0.25 mg kg^-1^ Cyto-D was administered by intrapleural injection 2 h before EV injection. The pleura of these mice was collected 24 h later and observed by stereomicroscopy (Nikon SMZ18, Tokyo, Japan).

### Degradation of EV proteins and RNAs

LLC-EVs were digested with 10 μg ml-1 protease K for 2 h with or without electroporation to degrade EV proteins. To degrade EV RNAs, LLC-EVs were digested with 10 μg ml^-1^ RNase I for 2 h in combination with electroporation. Electroporation of EVs was performed using a BTX electroporator (Harvard Biosciences, Cambridge, MA, USA). Briefly, 100 μg of LLC-EVs were mixed into 100 μl of electroporation buffer (Harvard Biosciences). An exponential program was performed at a fixed capacitance of 100 μF to obtain the optimum efficiency in 0.2 cm cuvettes.

### Isolation of p-pMCs

Murine p-pMCs were isolated based on a technique reported in a previous publication with some modifications 38. Briefly, mice were euthanized, and the thoracic walls were removed under sterile conditions. The parietal pleura was peeled off with ophthalmic forceps, washed twice with PBS to remove blood stains and cut into 1 mm^2^ tissue blocks. Then, pleural tissues were evenly inoculated in a 25 cm^2^ culture flask and grown in complete DMEM. Nonadherent cells were removed the next day, and MCs were ready for use after 72 h. Mesothelin on adherent and noadherent cells was detected by flow cytometry to confirm the isolation of p-pMCs.

### Immunohistochemical staining

Murine lung and human TT sections were routinely deparaffinized and rehydrated, and antigen retrieval was performed using 10 mM sodium citrate buffer (pH 6.0). After blocking with 5% BSA, slides were incubated with primary antibodies at 4 °C overnight and the corresponding HRP-conjugated secondary antibodies at RT for 30 min. Images were randomly acquired and analyzed using ImageJ software (NIH, Bethesda, MD, USA).

### Chemotaxis assay

For the DC chemotaxis assay, p-pMCs and 40L cells were stimulated with 2.5 μg ml^-1^ LLC-EVs and NCI-H2452 cells were stimulated with 2.5 μg ml^-1^ A549-EVs for 24 h. Then, the supernatants were collected and placed in the bottom wells of 3-μm pore-size Transwell chambers (Corning Inc., Corning, NY, USA) with DCs in the upper compartment for 12 h. DC migration was evaluated by counting the migrated cells via flow cytometry.

### Transfection of siRNAs and the miRNA mimic and inhibitor

Scrambled NC or targeting siRNA and the miRNA mimic, inhibitor or corresponding control (GenePharma, Shanghai, China) were transfected by using TransIT-TKO Transfection Reagent (Mirus Bio, Madison, WI, USA) according to the manufacturer's instructions. For *in vivo* siRNA delivery, 10 μg of cholesterol-conjugated siRNAs were dissolved in RNase-free water and intrapleurally injected into lung metastatic tumor-bearing mice 24 h before subsequent experiments. The siRNA, miRNA mimic and miRNA inhibitor sequences are listed in Table S5.

### Real-time PCR

Total RNA was extracted with TRIzol reagent (TaKaRa, Kusatsu, Shiga, Japan) and reverse transcribed into cDNA using a cDNA synthesis kit (Toyobo, Osaka, Japan). For miRNA reverse transcription, specific primers for the miRNAs of interest were synthesized by GenePharma. For relative quantitative analysis, real-time PCR was performed with SYBR Green (Vazyme, Nanjing, Jiangsu, China) on a Roche LightCycler® 480II instrument (Roche Diagnostics, Basel, Switzerland). The expression of the mRNAs of interest was normalized to GAPDH for cell and tissue samples and U6 for EV samples. Data were analyzed by the 2^-ΔΔCt^ method. For absolute quantitative analysis, real-time PCR was performed with specific TaqMan probes for the miRNAs of interest (GenePharma) and AceQ® Universal U^+^ Probe Master Mix V2 (Vazyme). To establish the standard curve of miRNA copy numbers and Ct values, 10-fold dilutions of synthetic miRNAs (GenePharma) were reverse transcribed and amplified. The absolute copy numbers of the miRNAs of interest were calculated according to the standard curve. A total of 10^8^ cel-miR-39-5p transcripts was used as the spike-in RNA control for normalization between samples. The primer and probe sequences are listed in Table S5.

### RNA-Seq analysis

Total RNA was isolated and then reverse transcribed into cDNA to generate an indexed Illumina library, sequenced at LC Sciences (Hangzhou, Zhejiang, China) on the HiSeq 2000 platform (Illumina). High-quality reads were aligned to the mouse reference genome (GRCm38) by Bowtie2, and the expression of individual genes was normalized to fragments per kilobase of exon model per million mapped reads data from RNA-Seq by expectation maximization. Then, the significant DEGs between PBS- and LLC-EV-treated 40L cells identified based on normalized deep sequencing counts were analyzed using the limma package with the |Log2FC | > 1 criteria and *P* value < 0.05. Subsequently, a heatmap of the DEGs was generated using the heatmap.2 function in the 'gplots' R package and Pearson correlation coefficients between pairs of DEGs were calculated with the 'corrplot' R package.

### MiRNA array

LLC-EVs and MLE-12-EVs were isolated as previously described and then dissolved in TRIzol. Then, they were sent for microarray analysis at Shanghai Biotechnology Co., Ltd. (Shanghai, China) using a SurePrint Mouse miRNA 8 × 60K v. 21.0 microarray (Agilent Technologies, Santa Clara, CA, USA). Feature Extraction Software v. 11.5.1.1 (Agilent Technologies) was used for acquisition, data extraction, and quality control analysis. After evaluating quality control parameters for each scanned microarray image, the data were extracted using Feature Extraction software. Gene Spring GX software 11.0 (Agilent Technologies) was used to normalize the signals. Then, the 'limma' R package was used to identify differentially expressed microRNAs with |Log2FC | > 1 criteria and *P* value < 0.05.

### Measurement of cytokine levels

P-pMCs and 40L cells were stimulated with 2.5 μg ml^-1^ LLC-EVs for 24 h. NCI-H2452 cells were stimulated with 2.5 μg ml^-1^ A549-EVs for 24 h. MPE fluid was centrifuged at 300 × g for 5 min to remove cells and debris. To detect cytokines secreted by p-pMCs, mice were euthanized, and equal areas of pleural tissues were cultured as described above for 24 h. The corresponding cytokine levels in culture supernatants and CCL21 levels in MPE fluid were measured by ELISA. To detect cytokines *in vivo,* serum from mice and humans were collected and levels of C1qA were measured by ELISA. Murine CCL19 (Absin, Shanghai, China), murine CCL21a and human Exodus 2 ELISA kits (Abcam, Cambridge, UK) and human C1qA ELISA kit (Abcam) were used in this study.

### ELISA measurement of EV miRNA levels

The same amounts of TT-EVs and MPE-EVs were captured on anti-CD63 antibody-coated 96-well ELISA plates by incubation overnight at 37 °C. After EVs were fixed and permeabilized with Intracellular Fixation and Permeabilization Buffer (Thermo Fisher Scientific) in RNase-free solution for 20 min (for miRNA detection), the plates were washed, and the miRNAs in the bound EVs were detected with 0.1 μg of biotinylated anti-CD9 antibodies or 20 nM biotin-labeled miR-5193 probe for 1 h at 37 °C. After being washed, 25 μl of avidin-HRP was added to individual wells and incubated for 1 h at RT. Subsequently, color was developed with TMB, the plates were blocked with 2 M H_2_SO_4_, and the absorbance was measured at 450 nm.

### Luciferase reporter assay

The WT mouse* Cd93* 3'-UTR luciferase reporter vectors were constructed by amplifying the mouse* Cd93* mRNA 3'-UTR containing the potential miR-5110 binding site and cloning it into the GP-miRGLO vector, a dual-luciferase reporter plasmid (Genepharma). The mutant C*d93* 3'-UTR was generated using the antisense strand of the WT sequence. 40L cells were cotransfected with the GP-miRGLO plasmid containing the WT *Cd93* 3'-UTR or mutant *Cd93* 3'-UTR and the indicated miRNA mimic or control (final concentration, 60 nM). After 24 h, cells were collected, and the luciferase activity in the cell lysates was measured with a Duo-Lite Luciferase Assay System (Vazyme).

### Generation of recombinant proteins and antibodies

Expression constructs for CD93 ligands were generated by cloning mouse C1qA, IGFBP7 and MMRN2 into the pcDNA3.1 vector with a flag tag. CD93 ligands were expressed by transiently transfecting 3T3 cells by JetPEI (Polyplus, Shanghai, China), and recombinant proteins with flag tags were isolated using anti-FLAG M2 magnetic beads (Sigma-Aldrich) and flag-tag peptides (Yeasen, Shanghai, China). Anti-mouse CD93 rabbit/mouse IgG1 chimeric mAb (Sino Biological, Beijing, China) was generated by cloning the variable region of antibodies from immunized rabbits and the constant region of mouse IgG1 antibodies into the pcDNA3.1 vector, and the clone M057 was selected as the most effective one to increase CCL21a level in p-pMCs.

### Endothelial tube formation assay

Normal lung tissues were digested with 2 mg ml^-1^ collagenase type I (Worthington Biochemical), 2 mg ml^-1^ collagenase type IV (Worthington Biochemical), and 0.2 mg ml^-1^ DNase I (Sigma-Aldrich) at 37 °C for 1 h, and terminated with culture medium containing 10% FBS. The single-cell suspension was obtained by filtering through a 70 μm cell strainer. CD31-positive primary ECs were isolated using anti-mouse CD31-PE antibodies (Biolegend, San Diego, CA, USA) and PE positive selection kit (StemCell, Vancouver, BC, Canada), and cultured in endothelial cell culture medium (Procell, Wuhan, China) for 5 days before use.

After treatment with 2 μg ml^-1^ IGFBP7 and/or 10 μg ml^-1^ M057 for 24 h, mouse primary ECs were seeded (1 × 10^4^ cells/well) in an ibidi plate precoated with endothelial growth factor-reduced Matrigel (BD Bioscience, Sab Jose, CA, USA). Mouse primary ECs were stained with 6.25 μg/ml calcein AM (PromoKine, Heidelberg, Baden‐Württemberg, Germany) for 15 min. Images for the capillary tubes were captured under an inverted fluorescence microscope and analyzed by ImageJ (NIH) angiogenesis analyzer plugins.

### Protein labeling and detection

According to the manufacturer's instructions, M057 was labeled with Protein Labeling Kits (Thermo Fisher Scientific). Then, 100 μg of Alexa Fluor 680-labeled anti-CD93 were intravenously injected into WT and *Cd93^-/-^* mice. After 24 h, the mice were sacrificed, and the bilateral chest walls were collected. The labeled anti-CD93 were imaged by an IVIS (PerkinElmer).

### Statistical analysis

All experiments are repeated at least twice, and all data are expressed as the mean ± s.d. values. Statistical analyses were performed with GraphPad Prism 8.0 software. Differences between the two groups were analyzed by unpaired Student's *t* test, and differences among multiple groups were analyzed by one-way ANOVA followed by Turkey's test. The Spearman rank-order correlation test was used for correlation analysis. A difference was considered significant if the *P* value was < 0.05.

## Supplementary Material

Supplementary figures and tables.

## Abbreviations

MCs: mesothelial cells
TEVs: tumor-derived extracellular vesicles
DCs: dendritic cells
pMCs: pleural MCs
LLC-EVs: EVs from murine LLC Lewis lung tumor cells
B16F10-EVs: EVs from murine B16F10 melanoma cells
VEGFR: vascular endothelial growth factor receptor
i.pl.: intrapleural
i.p.: intraperitoneal
s.c.: subcutaneous
TILs: tumor-infiltrating leukocytes
CD11c-DTR: CD11c-diphtheria toxin receptor
p-pMCs: primary pMCs
Cyto-D: Cytochalasin D
BMDCs: bone marrow-derived DCs
KD: knockdown
RNA-Seq: RNA sequencing
DEGs: differentially expressed genes
ACTD: Actinomycin D
HUVECs: human umbilical vein endothelial cells
MMRN2: multimerin-2
IGFBP7: insulin-like growth factor binding protein 7
CTLD: C-type lectin domain
ALT: alanine transaminase
AST: aspartate aminotransferase
αSMA: α-smooth muscle actin
NG2: neural/glial antigen 2
PFS: progression-free survival
