# Supplementary material for: Blockade of CD93 in pleural mesothelial cells fuels anti-lung tumor immune responses
Source: Theranostics. 2024 Jan 1;14(3):1010–28. doi: 10.7150/thno.89144 (PMC10797298; doi:10.7150/thno.89144)
Supplement: Supplementary file 1 — Supplementary figures and tables. [file thnov14p1010s1.pdf]

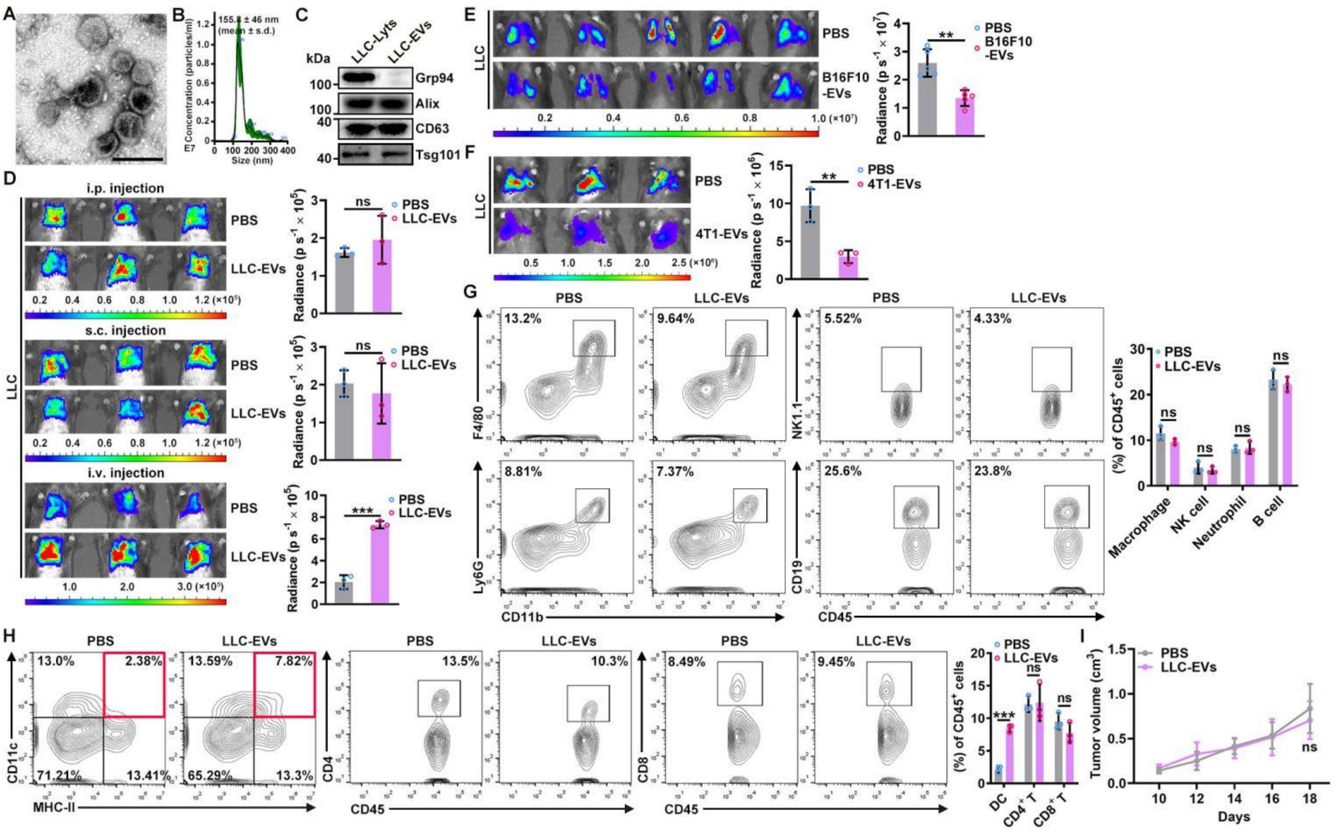

Figure S1. Intrapleurally injected TEVs suppress lung tumor growth by promoting DC recruitment. **A**, The morphology of LLC-EVs was evaluated by electron microscopy (EM). Scale bar, 100 nm. **B**, The size distribution of LLC-EVs was analyzed by nanoparticle tracking analysis (NTA). **C**, The indicated proteins in LLC-lyts and LLC-EVs were detected by western blotting. **D**, Mice were treated according to the protocol shown in Figure 1**A**, except that 5 μg LLC-EVs were injected via the indicated routes. The LLC-Luci lung tumor size was monitored with an IVIS on Day 30. **E**, **F**, Mice were treated according to the protocol shown in Figure 1**A**. LLC-Luci lung tumors were treated with 5 μg B16F10-EVs (**E**) or 4T1-EVs (**F**). The lung tumor size was monitored with an IVIS on Day 30. **G**, The indicated TIL subsets from the mice described in Figure 1**F** were detected by flow cytometry. **H**, **I**, Mice were subcutaneously inoculated with 1×10^6 LLC cells on Day 0 and intrapleurally injected with 5 μg LLC-EVs on Days 2,

4, 6, 8 and 10. DCs and T cells in the lungs were analyzed by flow cytometry on Day 18 (**H**), and the tumor size was measured (**I**). Representative results from three independent experiments are shown ($n = 3$, except for $n = 5$ in **E**, **I**). *$P < 0.05$; **$P < 0.01$; ***$P < 0.001$; ns, not significant (unpaired two-tailed Student's t test; mean and s.d.).

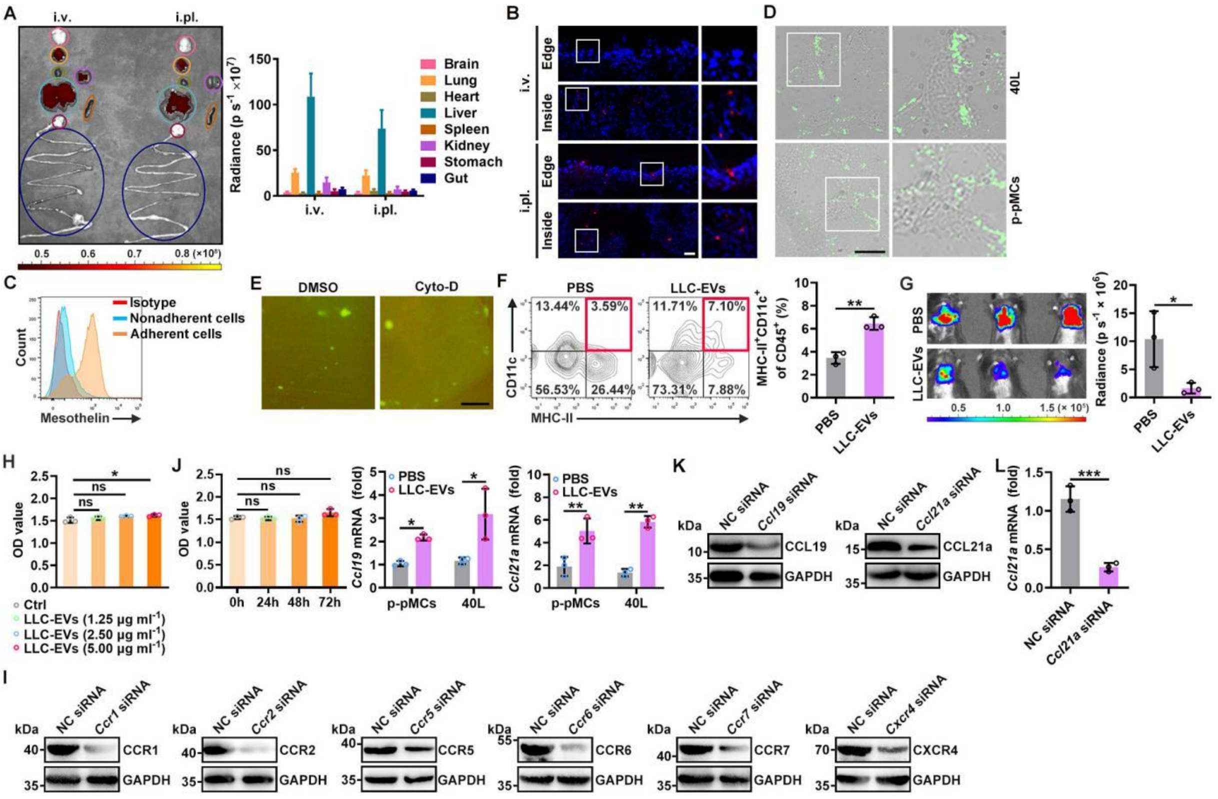

Figure S2. TEV-induced CCL21a secretion from pMCs promotes lung migration of DCs. **A**, Mice received i.v. or intrapleural injection of 100 μg VivoTrack 680-labeled (**A**) or 20 μg PKH26-labeled (**B**) LLC-EVs. Twenty-four hours later, the distribution of EVs in the indicated organs was detected with an IVIS (**A**), and that in the lungs was detected by fluorescence microscopy (**B**). **C**, Mesothelin on adherent (p-pMCs) and nonadherent cells isolated from pleura was detected by flow cytometry. **D**, P-pMCs and 40L cells were treated with 2.5 μg ml^{-1} CFSE-labeled LLC-EVs for 24 h, and uptake of EVs was detected by fluorescence microscopy. **E-G**, Mice were treated with LLC-Luci cells and CFSE-labeled LLC-EVs as shown in Figure 1E, except that 0.25 mg kg^{-1} Cyto-D was also intrapleurally injected 2 h after each EV injection. The incorporation of EVs into the pleura was detected by stereomicroscopy (**E**), the DC frequency among TILs was determined by flow cytometry (**F**), and the lung tumor size was monitored by

IVIS (**G**). **H**, 40L cells were stimulated with the indicated LLC-EV concentrations for 24 h. Cell viability was measured by a CCK8 assay. **I**, **K**, BMDCs (**I**) and 40L cells (**K**) were transfected with negative control (NC) siRNAs or the indicated targeting siRNAs, and the silencing efficiency was confirmed by western blotting. **J**, 40L cells were stimulated with 2.5 μg ml^{-1} LLC-EVs for 24 h, 48 h and 72 h. Cell viability was measured by a CCK8 assay. The *Ccl19* and *Ccl21*a mRNA levels in these cells were measured by real-time PCR. **L**, Mice were treated with intrapleural injection of 10 μg cholesterol-conjugated *Ccl21a* siRNAs or NC siRNAs for 24 h. Then, the parietal pleura was acquired from the above mice, and total RNA was extracted from 0.25 cm^2 pleural in 1 ml Trizol. The *Ccl21a* mRNA was measured by real-time PCR. Scale bar, 25 μm. Representative results from three independent experiments are shown (n = 3). *$P < 0.05$; **$P < 0.01$; ***$P < 0.001$; ns, not significant (unpaired two-tailed Student's *t* test; mean and s.d.).

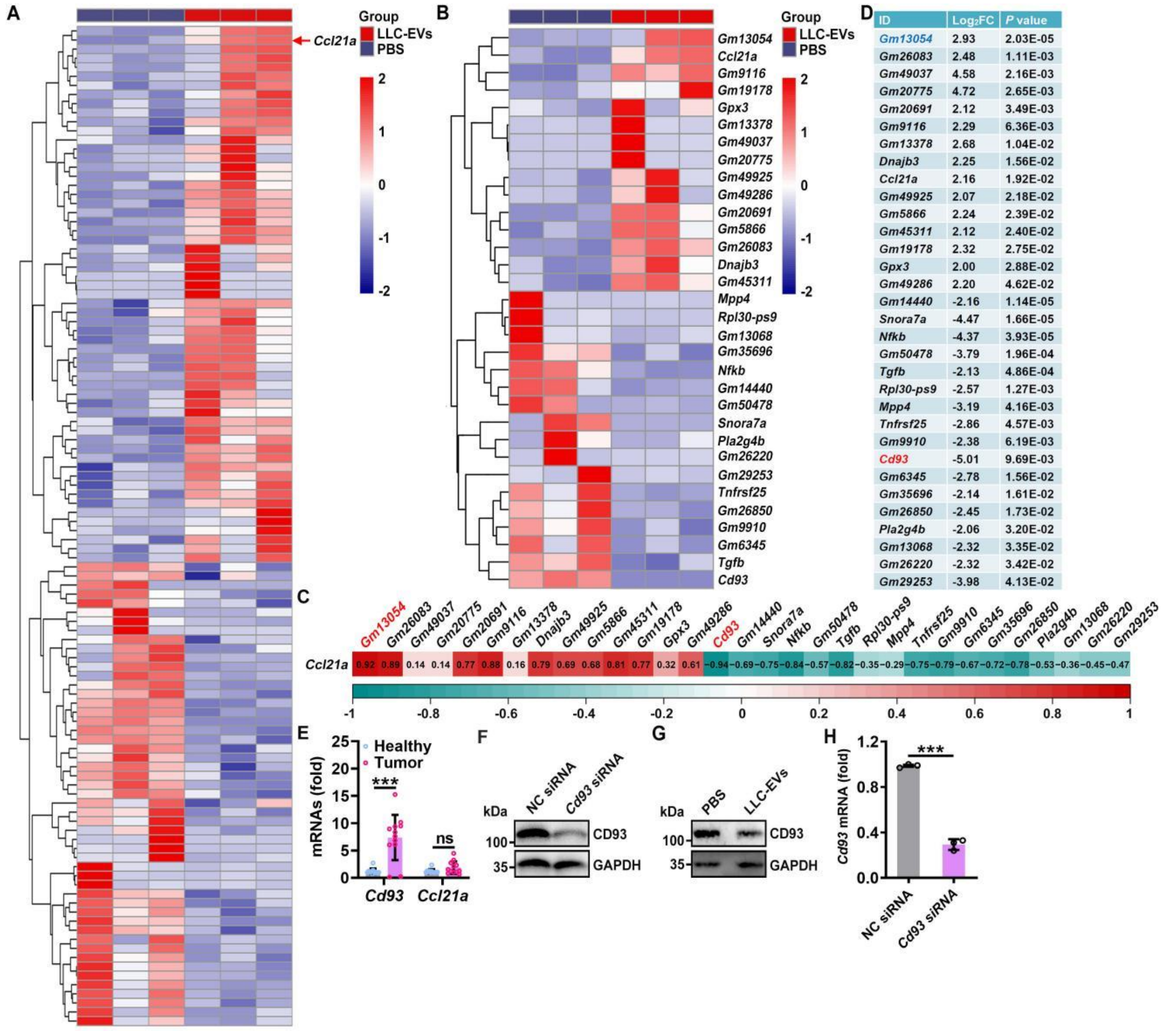

Figure S3. A decrease in CD93 level of pMCs induces CCL21a secretion by TEVs. **A**, **B**, mRNA levels in 40L cells with or without LLC-EV treatment were analyzed by RNA-Seq. All DEGs (**A**) and the DEGs with $|\text{Log}_2\text{FC}| \geq 2$ (**B**) are shown. **C**, The correlation of *Ccl21a* expression with that of the DEGs in **B** was analyzed. **D**, The indicated information for the DEGs in **B** is shown. **E**, The pleural *Cd93* and *Ccl21a* mRNA levels in healthy and LLC lung tumor-bearing mice were measured by real-time PCR. **F**, The silencing efficiency of CD93 in 40L cells was confirmed by western blotting. **G**, CD93 in p-pMCs with LLC-EV stimulation was detected by western blotting. **H**, Mice were treated with intrapleural injection of 10 μg cholesterol-conjugated *Cd93* siRNAs or NC siRNAs for 24 h. Then, the parietal pleura was

acquired from the above mice, and total RNA was extracted from 0.25 cm^2 pleural in 1 ml Trizol. The *Cd93* mRNA was measured by real-time PCR. Representative results from two independent experiments are shown ($n = 3$, except for $n = 12$ in **E**). ***$P < 0.001$; ns, not significant (unpaired two-tailed Student's *t* test; mean and s.d.).

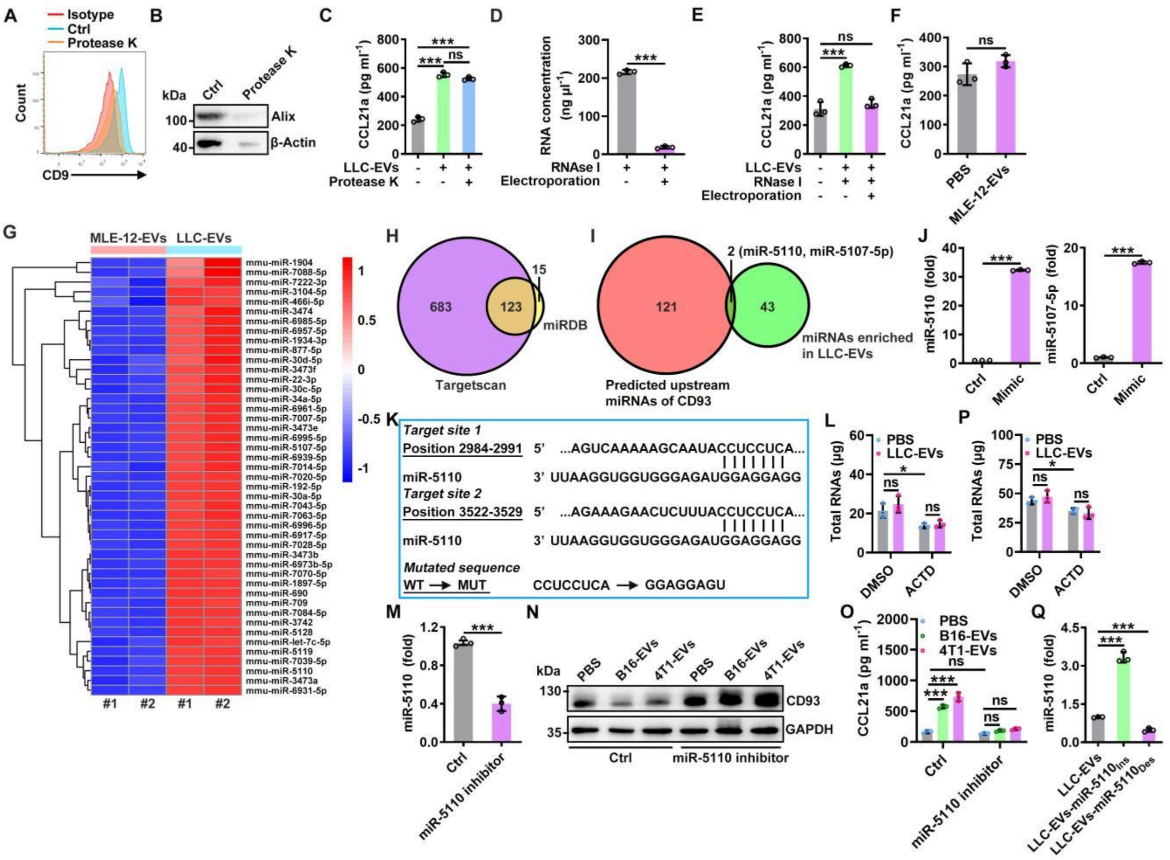

Figure S4. CD93 of pMCs is downregulated by TEV-derived miR-5110. **A-C**, LLC-EVs were digested with 10 μg ml^{-1} protease K for 2 h with electroporation. The digestion efficiency of the membrane-associated and internal proteins was confirmed by flow cytometry (**A**) and western blotting (**B**), as represented by CD9 (**A**) and Alix (**B**). 40L cells were stimulated with these EVs for 24 h, and the CCL21a levels in the supernatants of these cells were measured by ELISA (**C**). **D**, **E**, The RNA content in LLC-EVs digested with 10 μg ml^{-1} RNase I for 2 h was measured (**D**); 40L cells were stimulated with these EVs for 24 h, and CCL21a levels in the supernatants of these cells were measured by ELISA (**E**). **F**, 40L cells were stimulated with MLE-12EVs for 24 h, and the CCL21a levels in the supernatants of these cells were measured by ELISA. **G**, miRNAs in MLE-12-EVs and LLC-EVs were analyzed by a miRNA array approach; the enriched miRNAs in LLC-EVs are shown. **H**, The upstream miRNAs of *Cd93* were

predicted with the miRDB and TargetScan databases. **I**, Alignment of the enriched miRNAs and the predicted upstream miRNAs of CD93. **J**, Overexpression of miR-5110 or miR-5107-5p in 40L cells transfected with the indicated mimics for 24 h was confirmed by real-time PCR. **K**, The miR-5110 target sequence in the *Cd93* 3'-UTR and the corresponding mutated sequence are shown. **L**, 40L cells were stimulated with 2.5 μg ml^{-1} LLC-EVs for 24 h with or without 5 μg ml^{-1} ACTD. Then, total RNAs in these cells were isolated and quantified. **M**, Inhibition of miR-5110 in 40L cells transfected with the miR-5110 inhibitor was confirmed by real-time PCR. **N**, **O**, 40L cells transfected with the miR-5110 inhibitor were stimulated with 2.5 μg ml^{-1} B16F10-EVs and 4T1-EVs for 24 h. CD93 (**N**) and CCL21a (**O**) in these cells were detected by western blotting (**N**) and ELISA (**O**), respectively. **P**, Mice were intrapleurally injected LLC-EVs, along with or without 0.125 mg kg^{-1} ATCD injection 2 h ahead. Total RNAs in the pleura were isolated and measured by real-time PCR 8 h later. **Q**, miRNA-5110 levels in the indicated EVs were determined by real-time PCR. Representative results from three independent experiments are shown ($n = 3$). ***$P < 0.001$; ns, not significant (unpaired two-tailed Student's *t* test, except for one-way ANOVA followed by Tukey's test in **C**, **L**, **P**, **O**; mean and s.d.).

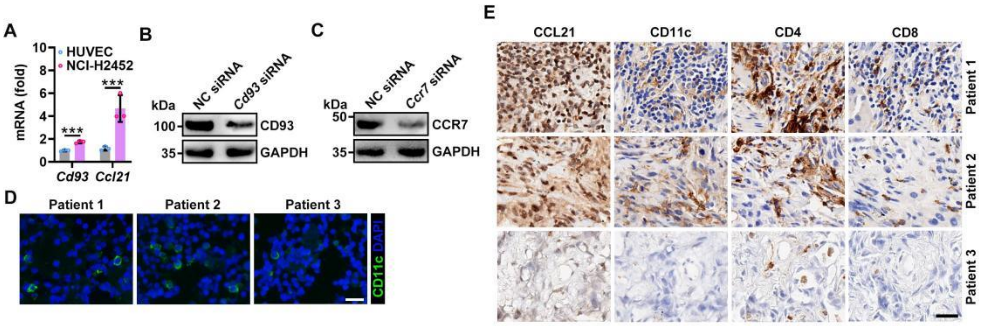

Figure S5. A decreased CD93 level of pMCs indicates increased T-cell responses in humans. **A**, *Cd93* and *Ccl21* mRNA levels in NCI-H2452 cells and HUVECs were measured by real-time PCR. **B**, **C**, The silencing efficiency of CD93 in NCI-H2452 cells (**B**) and CCR7 in DCs (**C**) was confirmed by western blotting. **D**, Representative immunofluorescence images of cell precipitates from MPEs. **E**, Representative immunohistochemical images of lung tumor tissues. Scale bar, 25 μm. Representative results from three independent experiments are shown (n = 3). ***P < 0.001; ns, not significant (unpaired two-tailed Student's t test; mean and s.d.).

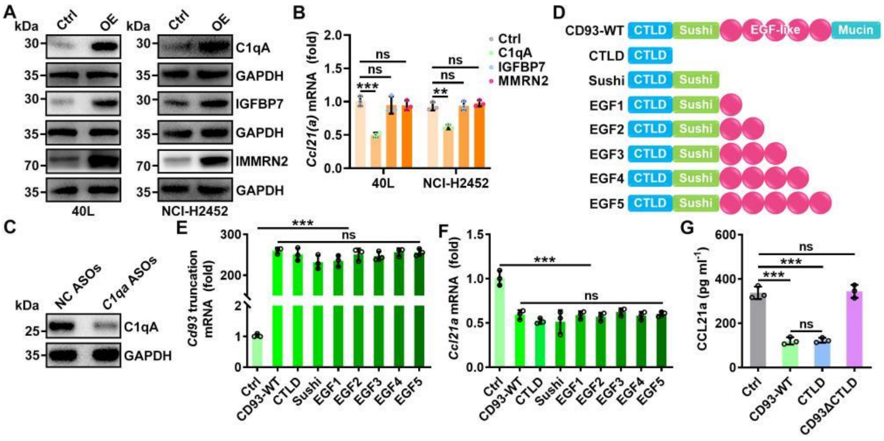

Figure S6. C1q is the ligand of CD93 responsible for regulating CCL21 in pleural MCs. **A**, overexpression of C1qA, MMRN2 and IGFBP7 in 40L or NCI-H2452 cells was confirmed by western blotting. **B**, *Ccl21a* mRNA levels in cells of **A** were measured by real-time PCR. **C**, The silencing effect of C1qA in the liver was confirmed by western blotting. **D**, Schematic of full-length and truncated CD93. **E**-**G**, 40L cells were transfected with the indicated truncations for 48 h. Levels of *Cd93* truncation (**E**) and *Ccl21a* mRNA (**F**) were measured by real-time PCR (**E**, **F**). CCL21a protein levels in the supernatants of these cells were measured by ELISA (**G**). Representative results from three independent experiments are shown (n = 3). **$P < 0.01$; ***$P < 0.001$; ns, not significant (one-way ANOVA followed by Tukey's test except for unpaired two-tailed Student's t test in **e**; mean and s.d.).

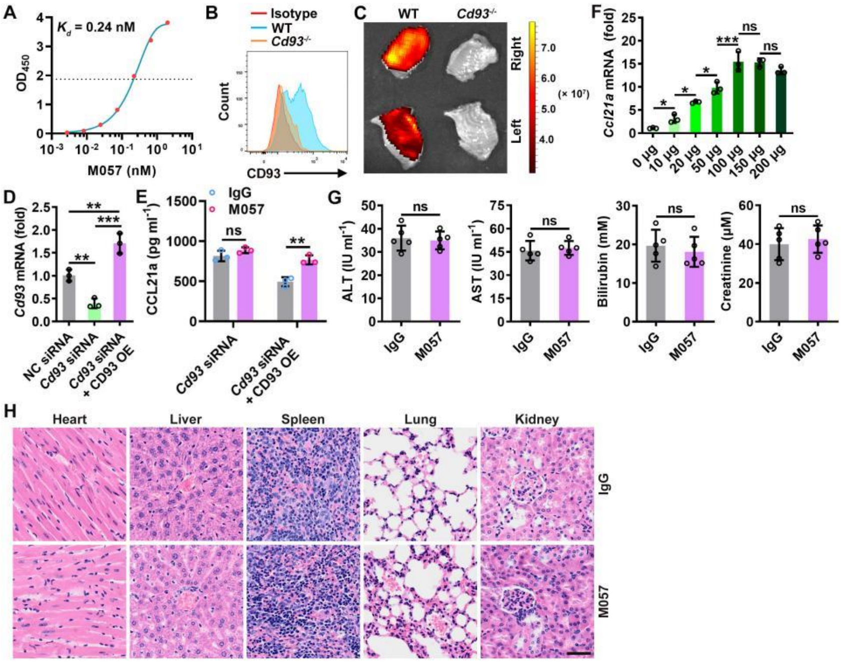

Figure S7. CD93 specific binding ani-CD93 has satisfying biosafety. **A**, The K_d of M057 binding to mouse CD93 was determined by ELISA. **B**, Staining of bone marrow cells from WT or *Cd93*$^{-/-}$ mice with M057. **C**, IVIS detection of WT or *Cd93*$^{-/-}$ murine pleura with i.v. injection of Alexa Fluor 680-labeled M057 for 24 h. **D**, **E**, 40L cells were transfected with *Cd93* siRNA for 24 h, along with or without CD93 overexpression for another 24 h. The *Cd93* mRNA levels in these cells were measured by real-time PCR (**D**). These cells were then treated with 10 μg ml^{-1} M057 for 24 h, and CCL21a in cell culture supernatants were measured by ELISA (**E**). **F**, LLC lung tumor-bearing mice were intravenously injected with the indicated doses of M057 for 24 h. The *Ccl21a* mRNA level in the pleura was measured by real-time PCR. **G**, **H**, Healthy mice were intravenously injected with 100 μg M057 every other day. The levels of ALT, AST, bilirubin and creatinine in sera were measured by ELISA (**G**), and histopathological damage in the heart, liver, spleen, lungs and kidneys was detected by H&E staining (Scale bar, 40 μm) (**H**) after the fifth injection.

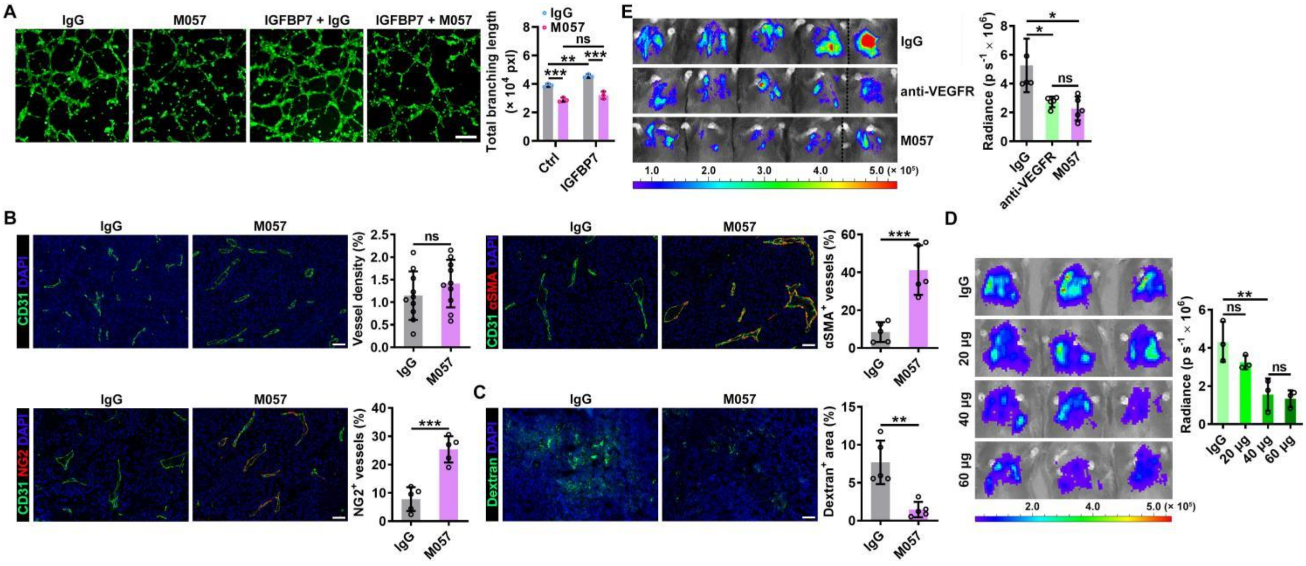

Figure S8. CD93 specific binding ani-CD93 has satisfying biosafety. **A**, Mouse primary ECs were treated with 10 μg ml^{-1} M057 for 24 h in the presence of 2 μg ml^{-1} IGFBP7 followed by calcein-AM staining. Then, tube formation was detected by fluorescence microscopy and statistically analyzed. **B**, **C**, LLC lung tumor-bearing mice were intravenously injected with 100 μg M057 on Days 14, 16, 18, 20 and 22. Tumor tissues were collected and stained with NG2 and CD31 or αSMA and CD31 and quantitatively analyzed (**B**), or tumor endothelial permeability was assessed by perfusion with 5 mg FITC-dextran (70 kDa) (**C**) on Day 25. **D**, LLC-Luci lung tumor-bearing mice were intravenously injected with serial doses of anti-VEGFR Days 14, 16, 18, 20 and 22. Lung tumor size was monitored with an IVIS on Day 25. **E**, LLC lung tumor-bearing *Ccr7*$^{-/-}$ mice were intravenously injected with 40 μg anti-VEGFR or 100 μg M057 Days 14, 16, 18, 20 and 22. Lung tumor size was monitored with an IVIS on Day 25. Representative results from three independent experiments are shown (n = 3).

Scale bar, 50 µm. *$P < 0.05$; **$P < 0.01$; ***$P < 0.001$ (one-way ANOVA followed by Tukey's test in **A**, **D**, **E**; unpaired two-tailed Student's *t* test in **B**, **C**; mean and s.d.).

Table S1: Clinical characteristics of patients with MPE (n = 33)

**Characteristics**	**Number of patients**
**Gender**	
Male	17
Female	16
**Age**	
≤ 50	2
50-75	24
> 75	7
**Histological subtype**	
Adenocarcinoma	25
Squamous cell carcinoma	8
**History of therapy**	
Surgery	1
Chemotherapy	19
None	13

Table S2: Clinical characteristics of lung cancer patients (n = 73)

**Characteristics**	**Number of patients**
**Gender**	
Male	40
Female	33
**Age**	
≤ 50	7
50-75	65
> 75	1
**Histological subtype**	
Adenocarcinoma	45
Squamous cell carcinoma	26
Adenosquamous carcinoma	2

Table S3: Clinical characteristics of lung cancer patients with anti-PD-1 therapy (n = 60)

**Characteristics**	**Number of patients**
**Gender**	
Male	57
Female	3
**Age**	
≤ 50	1
50-75	42
> 75	17
**Histological subtype**	
Adenocarcinoma	14
Squamous cell carcinoma	44
Large cell carcinoma	2
**PD-L1 expression**	
＜1%	13
1-50%	23
⩾50%	20
Unknown	4
**αPD-1 preparations used for treatment**	
Pembrolizuma	9
Nivolumab	4
Tislelizumab	22
Sintilimab	16
Camrelizumab	4
Durvalumab	1
Toripalimab	4

Table S4: The information of antibodies used in this study

Antibodies	Resource	Identifier	Dilution ratio
Fixable Viability Dye eFluor 450	eBioscience	Cat# 65-0863-14	1:500
Fixable Viability Dye eFluor 520	eBioscience	Cat# 65-0867-14	1:500
anti-mouse CD45 PB	BioLegend	Cat# 103126	1:500
anti-mouse CD45.2 APC	eBioscience	Cat# 17-0454-82	1:500
anti-mouse CD45.1 FITC	eBioscience	Cat# 11-0453-81	1:500
anti-mouse CD4 PE	eBioscience	Cat# 12-0041-83	1:500
anti-mouse CD8α APC	BioLegend	Cat# 100712	1:500
anti-mouse MHCII APC	eBioscience	Cat# 17-5321-82	1:500
anti-mouse CD11c PE	eBioscience	Cat# 12-0114-82	1:500
anti-mouse CD11b PE	eBioscience	Cat# 12-0112-83	1:500
anti-mouse F4/80 APC	BioLegend	Cat# 123116	1:500
anti-mouse Ly6G APC	BioLegend	Cat# 127641	1:500
anti-mouse NK1.1 PE	eBioscience	Cat# 12-5941-83	1:500
anti-mouse CD19 PE	eBioscience	Cat# 12-0193-82	1:500
anti-mouse CCR7 PE	eBioscience	Cat# 12-1979-42	1:500
anti-mouse biotin-conjugated CD8α	eBioscience	Cat# 13-0081-85	3 μg ml^{-1}
anti-mouse biotin-conjugated CD9	BioLegend	Cat# 124804	1:125
purified anti-mouse CD63	BioLegend	Cat# 353039	1:125
anti-mouse/human CD4	Abcam	Cat# ab288724	1:1000 (IHC); 1:200
anti-mouse CD8α	Abcam	Cat# ab217344	1:200
anti-human CD8α	Abcam	Cat# ab237709	1:1000
anti-mouse CD11c	Abcam	Cat# ab219799	1:200
anti-human CD11c	Abcam	Cat# ab52632	1:1000
anti-mouse Mesothelin	Abcam	Cat# ab236546	1:200
anti-mouse/human CD93	Affinity	Cat# DF8338	1:1000 (WB); 1:200 (IF)
anti-mouse/human CCL21	Affinity	Cat# DF6681	1:200
anti-mouse/human CD81	Affinity	Cat# DF2306	1:1000
anti-mouse/human Grp94	Abclonal	Cat# A0989	1:1000
anti-mouse/human Alix	Abclonal	Cat# A2215	1:1000
anti-mouse/human CD63	Abclonal	Cat# A19023	1:1000
anti-mouse/human Tsg101	Abclonal	Cat# A1692	1:1000
anti-mouse/human Hsp70	Abclonal	Cat# A0284	1:1000
anti-human Hemoglobin A1	Abclonal	Cat# A9293	1:1000
anti-mouse CCL19	Abclonal	Cat# A16972	1:1000
anti-mouse CCR1	Abclonal	Cat# A18341	1:1000
anti-mouse CCR2	Abclonal	Cat# A2855	1:1000
anti-mouse CCR5	Abclonal	Cat# A20261	1:1000
anti-mouse CCR6	Abclonal	Cat# A16206	1:1000

anti-mouse CCR7	Abcam	Cat# ab32527	1:2000
anti-mouse CXCR4	Proteintech	Cat# 11073-2-AP	1:1000
anti-mouse/human C1qA	Proteintech	Cat# 11602-1-AP	1:1000
anti-mouse/human IGFBP7	Proteintech	Cat# 19961-1-	1:1000
anti-mouse MMRN2	Abmart	Cat# PK59393S	1:1000
anti-human MMRN2	Absin	Cat# abs101735	1:1000
anti-mouse/human GAPDH	Proteintech	Cat# 60004-1-Ig	1:1000
anti-mouse CD31	Proteintech	Cat# 66065-2-Ig	1:200
anti-mouse αSMA	Proteintech	Cat# 67735-1-Ig	1:200
anti-mouse NG2	Proteintech	Cat# 55027-1-	1:200
anti-mouse CD4	BioXcell	Cat# BP0003-1	60 µg every two days
anti-mouse CD8α	BioXcell	Cat# BP0061	16 µg every two days
anti-mouse VEGFR2	BioXcell	Cat# BP0060	40 µg every two days
Goat anti-mouse IgG HRP	MultiSciences	Cat# GAM0072	1:1000
Goat anti-rabbit IgG HRP	MultiSciences	Cat# GAR0072	1:1000
anti-Rabbit IgG H&L (Alexa Fluor 488)	MultiSciences	Cat# GAR4882	1:200
anti-Mouse IgG H&L (Alexa Fluor 488)	MultiSciences	Cat# GAM4882	1:200
anti-Rabbit IgG H&L (Alexa Fluor 594)	MultiSciences	Cat# GAR5942	1:200

Abbreviations: IHC, immunohistochemistry; IF, immunofluorescence; WB, western blotting.

Table S5: The sequences of oligonucleotides	
**Primers for qPCR**	
**Name**	**Sequence (5’ to 3’)**
mCcl21a-F	GTGATGGAGGGGGTCAGGA
mCcl21a-R	GGGATGGGACAGCCTAAACT
mCd93-F	ATCTCAACTGGTTTGTTCCTGC
mCd93-R	ACTCTTCACGGTGGCAAGATT
mGAPDH-F	CATCACTGCCACCCAGAAGACTG
mGAPDH -R	ATGCCAGTGAGCTTCCCGTTCAG
mmu-miR-5110-F	CAGATGGAGGAGGTAGAGGGTG
mmu-miR-5110-R	GTGCAGGGTCCGAGGT
mmu-miR-5107-5p-F	GAGACTTGTGGGCAGAGGAGG
mmu-miR-5107-5p-R	TATGGTTGTTGACGACTGGTTGAC
U6-F	CAGCACATATACTAAAATTGGAACG
U6-R	ACGAATTTGCGTGTCATCC
hsa-miR-5193-F	CGACGACTCCTCCTCTACCTCAT
hsa-miR-5193-R	TATGGTTGTAGAGCAGTGGTTGAC
**MiRNA mimics and inhibitors**	
**Name**	**Sequence (5’ to 3’)**
mmu-miR-5110 mimic	GGAGGAGGUAGAGGGUGGUGGAAUU
mmu-miR-5107-5p mimic	UGGGCAGAGGAGGCAGGGACA
miRNA mimic NC	UUCUCCGAACGUGUCACGUTT
mmu-miR-5110 inhibitor	AAUUCCACCACCCUCUACCUCCUCC
mmu-miR-5107-5p inhibitor	UGUCCCUGCCUCCUCUGCCCA
miRNA inhibitor NC	CAGUACUUUUGUGUAGUACAA
**siRNAs for gene knockdown**	
**Name**	**Sequence (5’ to 3’)**
mCcr1 siRNA F	CCCUGUAUUCUCUAGUGUUUAdTdT
mCcr1 siRNA R	UAAACACUAGAGAAUACAGGGdTdT
mCcr2 siRNA F	AGGUCUCGGUUGGGUUGUAAAdTdT
mCcr2 siRNA R	UUUACAACCCAACCGAGACCUdTdT
mCcr5 siRNA F	CCAUGCUGUGUUUGCUUUAAAdTdT
mCcr5 siRNA R	UUUAAAGCAAACACAGCAUGGdTdT
mCcr6 siRNA F	CCCGUGUUGUAUGCGUUUAUUdTdT
mCcr6 siRNA R	AAUAAACGCAUACAACACGGGdTdT
mCcr7 siRNA F	AGGGCAUCUUUGGCAUCUAUdTdT
mCcr7 siRNA R	UAUAGAUGCCAAAGAUGCCCUdTdT
mCxcr4 siRNA F	GCCUGCUGGCUGCCAUAUUAUdTdT

mCxcr4 siRNA R	AUAAUAUGGCAGCCAGCAGGCdTdT
mCcl19 siRNA F	GAAGUCUUCUGCCAAGAACAAdTdT
mCcl19 siRNA R	UUGUUCUUGGCAGAAGACUUCdTdT
mCcl21a siRNA F	AGGAAGCAAGAACCAAGUUUAdTdT
mCcl21a siRNA R	UAAACUUGGUUCUUGCUUCCUdTdT
mCd93 siRNA F	CUCCUGUAAAGAGGGCUAUAUdTdT
mCd93 siRNA R	AUAUAGCCCUCUUUACAGGAGdTdT
hCd93 siRNA F	CGAGACUCAGAGUCAUUAUUUdTdT
hCd93 siRNA R	AAAUAAUGACUCUGAGUCUCGdTdT
hCcl21 siRNA F	CACUCUUUCUCCUGCUUUAACdTdT
hCcl21 siRNA R	GUUAAAGCAGGAGAAAGAGUGdTdT
hCcr7 siRNA F	UACACUUUGUUCGAGUCUUUGdTdT
hCcr7 siRNA R	CAAAGACUCGAACAAAGUGUAdTdT
NC siRNA F	UUCUCCGAACGUGUCACGUdTdT
NC siRNA R	ACGUGACACGUUCGGAGAAdTdT
mC1qa ASO	ATTGCCTGGATTGCCTTTC
NC ASO	CAGTACTTTTGTGTAGTACAA
**Taqman probe**	
**Name**	**Sequence (5’ to 3’)**
mmu-miR-5110 Taqman probe	CATCCCTATCCAACCATACAGACAATTCCAC
hsa-miR-5193 Taqman probe	CCTATCCAACCATACAGACACTGGG
**miRNA detection probe**	
**Name**	**Sequence (5’ to 3’)**
mmu-miR-5110 probe	AATTCCACCACCCTCTACCTCCTCC
hsa-miR-5193 probe	ACTGGGATGAGGTAGAGGAGGA
